# Nutritional Composition, Therapeutic Benefits, and Functional Food Potential of *Coleus amboinicus*: A Comprehensive Review

**DOI:** 10.1002/fsn3.71419

**Published:** 2026-06-08

**Authors:** Shani Upadhyay, Rakesh Verma, Shikha Gupta

**Affiliations:** ^1^ Department of Pharmacology, IMS BHU Varanasi Uttar Pradesh India; ^2^ MMLT, Rajiv Gandhi South Campus BHU Mirzapur Uttar Pradesh India

**Keywords:** bioactive compounds, *Coleus amboinicus*, food preservation, functional food, nutritional composition, therapeutic potential

## Abstract

*Coleus amboinicus* (Lour.), commonly called Indian borage, Mexican mint, and Oregano thyme, is a member of the Lamiaceae family, native to Southeast Asia and Africa. This herb is widely known for its rich nutritional composition, including essential vitamins, minerals, and bioactive compounds such as thymol, carvacrol, and rosmarinic acid. The therapeutic potential of *C. amboinicus* lies in its antioxidant, anti‐inflammatory, antimicrobial, and prebiotic properties, which aid in managing chronic diseases such as cardiovascular issues, diabetes mellitus, and respiratory conditions. Recent studies have demonstrated its potential in food preservation, offering a natural alternative to synthetic preservatives due to its antimicrobial properties. Despite its promising applications, challenges such as variability in bioactive content, a lack of robust clinical trials, and regulatory approval remain. This review examines the nutritional composition, therapeutic benefits, and potential as a functional food of *C. amboinicus* . It suggests future research directions to overcome existing barriers and enhance integration into modern dietary systems.

## Graphical abstract

*Coleus amboinicus* is a nutrient‐rich herb containing vitamins (A‐, C‐, B‐complex), minerals (Ca, Mg, Fe, K), dietary fiber, and potent bioactives such as rosmarinic acid, quercetin, thymol, and carvacrol. These compounds contribute to antioxidant, anti‐inflammatory, antimicrobial, prebiotic, and cardioprotective benefits. With its strong nutritional profile and therapeutic phytochemistry, *C. amboinicus* shows high potential as a functional food ingredient, natural preservative, and nutraceutical agent for modern health‐oriented food systems.

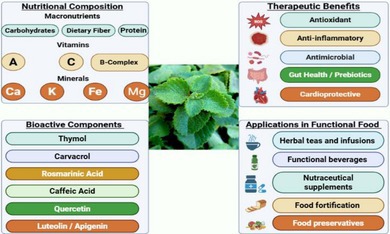

## 1 Introduction

Functional foods have gained significant popularity in recent years as consumers increasingly seek natural and plant‐based nutritional products to address lifestyle‐related health issues. These foods provide health benefits beyond basic nutrition, aiding in the prevention or management of conditions like cardiovascular diseases, diabetes, obesity, and inflammation (Yu et al. 2025). As demand for functional foods increases, plants rich in bioactive compounds have become a major area of research and development. Among them, *Coleus amboinicus* (Lour.), also known as Indian borage, Mexican mint, or oregano thyme, has emerged as a valuable herb with potential applications in modern food systems. *C. amboinicus* belongs to the mint family (Lamiaceae), which also includes other medicinally important herbs such as basil, rosemary, and thyme. This perennial herb, native to Southeast Asia and Africa, is now cultivated worldwide for its culinary, medicinal, and nutritional properties (Granato et al. 2020; Serafini and Peluso 2017). The plant's unique aroma and flavor, attributed to its high essential oil content, make it a popular culinary herb in traditional cuisines. Moreover, its widespread use in conventional medicine highlights its therapeutic importance. *C. amboinicus* has also been used as a spice for centuries and is commonly employed to treat respiratory, digestive, and skin disorders. Its leaves can be applied as a poultice for wounds, brewed into tea to relieve respiratory issues, or used as a remedy for colds and coughs (Paul et al. 2024; Ślusarczyk et al. 2021). The plant exhibits characteristic Lamiaceae features, including a fleshy, aromatic stem, pubescent ovate leaves with crenate margins, and bilabiate flowers arranged in terminal racemes, as summarized in Table 1.

**TABLE 1:** Morphological features of *Coleus amboinicus*.

Morphological feature	Description
Family	Lamiaceae
Common name	Indian Borage, Mexican Mint, Cuban Oregano
Habit	Perennial, succulent, aromatic herb
Stem	Thick, fleshy, and highly branched; square‐shaped with a slightly hairy surface
Leaves	Opposite, ovate, fleshy, and pubescent (hairy); margins are crenate (scalloped); have a strong aromatic odor.
Leaf color	Green, sometimes tinged with purple at the edges.
Flowers	Small, bilabiate (two‐lipped), pale purple to bluish, arranged in terminal racemes
Roots	The fibrous root system sometimes produces adventitious roots.
Fruits	Very rare; when present, they are nutlets
Odor	Strongly aromatic, similar to oregano or thyme

**FIGURE 1:**
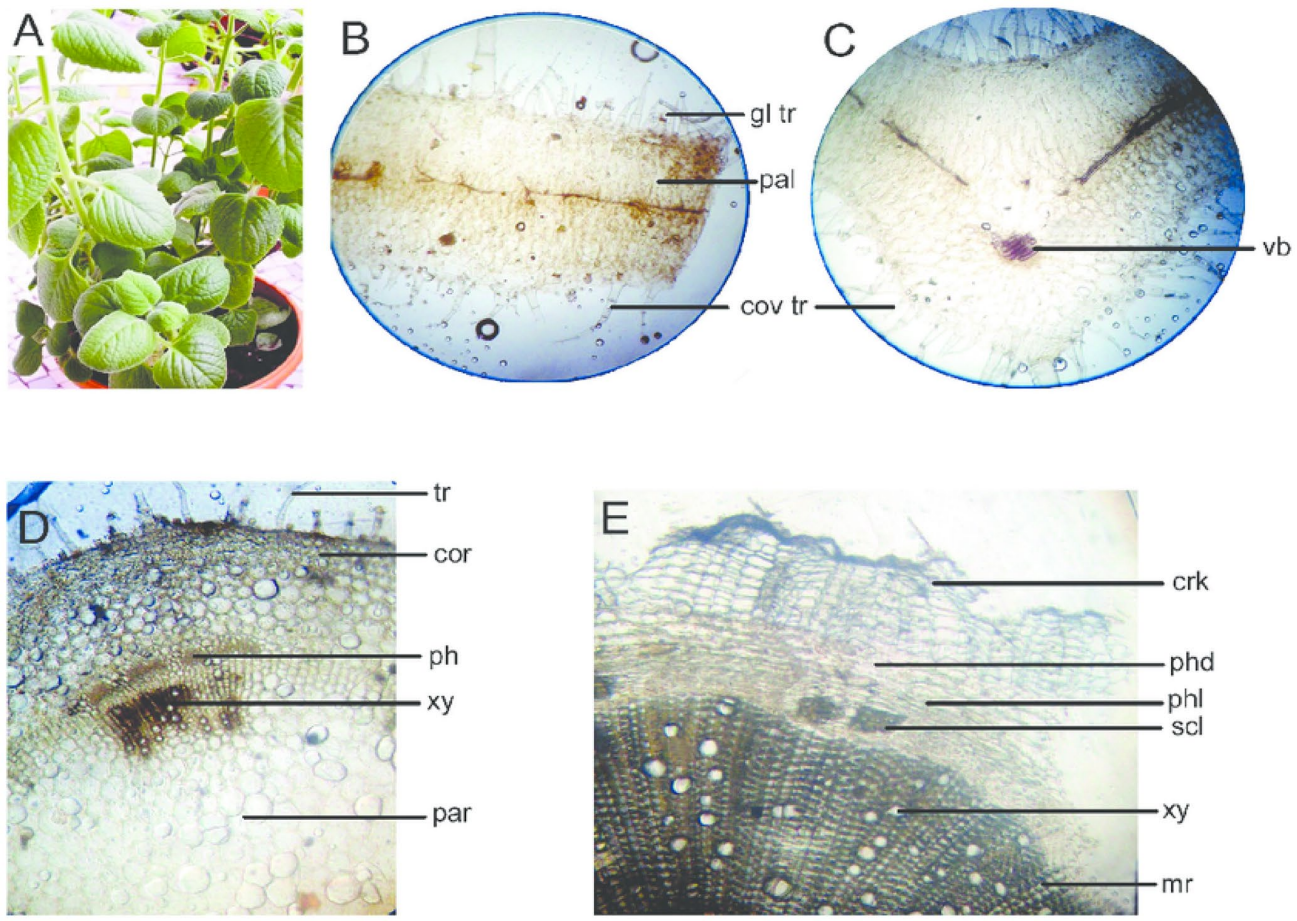
Morpho‐anatomical features of *Coleus amboinicus* . (A) The plant was observed in its native habitat, featuring fleshy, thick leaves with an acute apex, dentate edges, and a symmetrical base, as well as smooth, flexible, succulent stems with multiple nodes and internodes. (B) A transverse slice of the leaf lamina reveals glandular trichomes, covering trichomes, and well‐developed palisade cells. (C) A transverse slice of the midrib reveals the primary vascular bundle. (D) A transverse slice of the stem reveals trichomes, cortex, phloem, xylem, and parenchyma. (E) Transverse root segment displaying cork, phellogen, phelloderm, sclerenchyma, phloem, xylem, and medullary rays. Roots are cylindrical or torus‐shaped, with conspicuous rootlets. This figure is reproduced from Hullatti and Bhattacharjee (2011), *Pharmacognosy Journal*, 3 (24): 39–44, under the CC BY‐NC‐ND 4.0 license.

The morpho‐anatomical analysis of *C. amboinicus* , shown in Figure 1, reveals distinct structural features, confirming its taxonomic position in the Lamiaceae. Its leaf is typically dorsiventrally organized, having clearly differentiated palisade and spongy mesophyll with a central vascular bundle that supports effective transportation of water and nutrients. The general view of the epidermal surface shows a high density of both glandular and non‐glandular trichomes, indicating active secretion of volatile and phenolic metabolites and helping create a mechanical barrier against environmental stresses. The general structure of the stem features a well‐developed cortex followed by a continuous vascular ring of phloem and xylem tissues, with the surrounding ground tissue parenchyma being highly developed for storage and metabolic activities. At the junction between the root and stem, mature tissues exhibit clear secondary thickening with visible layers of cork and periderm, along with reinforcing sclerenchyma and regular medullary rays, indicating perennial traits and mechanical stability. These morpho‐anatomical features serve as reliable diagnostic markers for authentication. They are structurally relevant to the biosynthesis, accumulation, and storage of bioactive compounds important for nutritional and functional food applications (Hullatti and Bhattacharjee 2011).

Scientific interest in *C. amboinicus* has grown significantly due to its diverse phytochemical profile. The herb is rich in essential oils, including thymol and carvacrol. Additionally, it contains phenolic acids, such as rosmarinic acid, along with flavonoids like quercetin, as well as various vitamins and minerals (Paul et al. 2024). Collectively, these compounds contribute to its antioxidant, anti‐inflammatory, antimicrobial, and immune‐enhancing properties, making it an attractive candidate for the development of functional foods (Kim et al. 2022). With the growing global interest in natural ingredients, these properties position *C. amboinicus* as a promising option for promoting health, particularly in individuals with chronic conditions or disabilities. *C. amboinicus* is a versatile herb used in dietary supplements, herbal teas, and fortified foods.

Besides its bioactive properties, the nutritional content of *C. amboinicus* makes it suitable for daily consumption. The plant is an excellent source of key micronutrients, including dietary fiber, protein, calcium, magnesium, potassium, and vitamins A and C (Sabra et al. 2018; Sawant et al. 2023). Its rich fiber content supports gut health by promoting beneficial microflora and enhancing digestion, while its mineral composition helps maintain bone health and cardiovascular function (Ashaari et al. 2021; Silva et al. 2017; Phattayanon et al. 2025; Sajimin et al. 2025).

The increasing prevalence of chronic diseases worldwide highlights the need for nutritional interventions that combine therapeutic and nutritional values. *C. amboinicus* offers dual benefits as both a medicinal plant and a functional food ingredient. Recent studies have explored its potential to address specific health issues, including oxidative stress, hypertension, and microbial infections (Sabra et al. 2018; Sawant et al. 2023). These findings support the incorporation of *C. amboinicus* into innovative food products, promoting public health. Although *C. amboinicus* shows great promise, several obstacles hinder its widespread application. Variations in cultivation practices, extraction methods, and environmental conditions lead to inconsistent levels of bioactive compounds, making standardization challenging. Moreover, the lack of robust clinical studies on its efficacy and safety presents significant barriers to regulatory approval (Ashaari et al. 2021; Silva et al. 2017). Overcoming these obstacles requires a multidisciplinary approach that encompasses agricultural practices, food science, pharmacology, and collaboration with regulatory agencies. This review aims to provide a comprehensive overview of the nutritional composition of *C. amboinicus* and its benefits as a functional food. It highlights the bioactive properties of *C. amboinicus* , its applications in food systems, and the challenges associated with commercialization. By identifying future research directions, this paper aims to establish *C. amboinicus* as a valuable component of modern nutritional interventions.

The agricultural yield of *C. amboinicus* plants depends on climate, soil quality, and farming practices. In tropical and subtropical regions, these plants exhibit vigorous vegetative growth and can be harvested multiple times annually. Field studies in India and Indonesia have shown an average annual fresh biomass yield of 18 to 25 tons per hectare, with dry leaf yields of approximately 3 to 5 tons per hectare under typical farming methods and moderate irrigation. In controlled pot‐culture experiments, a 60‐day harvest interval yielded the highest shoot dry‐matter production of approximately 34 g per plant, highlighting the significance of harvest timing on productivity. Leaf essential oil content ranges from 0.07% to 1.62%, corresponding to an average oil yield of 8–15 L ha^−1^, which may exceed 100 L ha^−1^ when optimal fertilization and irrigation strategies are applied to this crop. In India, *C. amboinicus* is cultivated mainly in the southern states of Tamil Nadu, Kerala, and Karnataka as a small‐scale medicinal and aromatic crop. According to the Indian Institute of Horticultural Research, the perennial plant can be harvested for 2 years. This produces successive flushes of foliage and essential oil following every pruning cycle (ICAR‐IIHR 2021; Sabra et al. 2018; Silva et al. 2017; Sajimin et al. 2025; Rohini and Smitha 2021).

The usage of *C. amboinicus* and its derivatives in the herbal, nutraceutical, and essential oil industries has increased their market worth in recent years. Exporters India (2025) estimates that dried leaf powder fetches around ₹800–₹1000 kg^−1^ (US$9–US$12 kg^−1^) in the Indian herbal raw material market, notwithstanding the lack of species‐specific economic figures at present. Globally, *C. amboinicus* is classify 2QAWSEDRFTYUed alongside *Coleus* and turmeric, with a market value of approximately US $3.75 billion in 2024 and an estimated CAGR of about 5.6% through 2035. The growing need for natural preservatives, fragrant herbs, and phytotherapeutic components has established *C. amboinicus* as a prospective commercial crop in the medicinal plant industry. *C. amboinicus* ' agricultural productivity and commercial viability in both local and global markets can be increased by the adoption of standardized cultivation techniques combined with large‐scale essential oil extraction and value‐added product formulation (Exporters India 2025; MRF 2025).

## 2 Nutritional Composition of *Coleus amboinicus*

**FIGURE 2:**
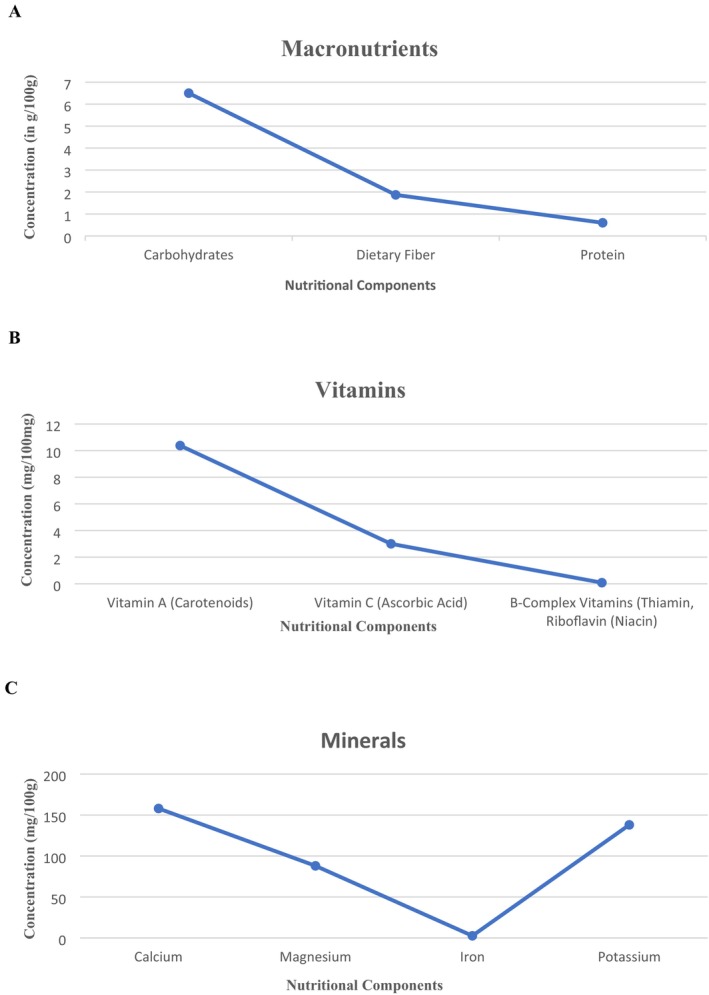
The image shows the nutritional profile of *Coleus amboinicus* , including Graph A (macronutrients: 7 g carbohydrates, 1.87 g fiber, 0.6 g protein/100 g), Graph B (vitamins: 10.38 mg vitamin A, 3 mg vitamin C, 0.08 mg thiamine/100 g), and Graph C (minerals: 158 mg calcium, 88 mg magnesium, 2.62 mg iron, 138 mg potassium/100 g). These graphs highlight its nutrient‐dense composition.

*C. amboinicus* is a nutritionally valuable and functionally important plant. Rich in bioactive compounds, vitamins, minerals, and macronutrients, it has been extensively studied for its potential in addressing modern health issues. The distribution of macronutrients, vitamins, and minerals is graphically represented in Figure 2. This section reviews the nutritional composition and synthesizes findings from various studies to highlight its significance in functional foods and therapeutic applications (Paul et al. 2024; El‐hawary, El‐sofany, Abdel‐Monem, and Ashour 2012). The detailed nutritional profile of *C. amboinicus* is presented in Table 2.

**TABLE 2:** Nutritional composition of *Coleus amboinicus*.

Category	Nutrient/compound	Concentration/presence	Health benefits	Applications	References
Macronutrients	Carbohydrates (complex)	Estimated 6–6‐7 g/100 g (fresh leaves) 61.59 g/100 g (in biscuits)	Provides sustained energy, stabilizes blood sugar	Energy supplements and balanced diets	(Serafini and Peluso 2017; Phattayanon et al. 2025; Sajimin et al. 2025; Thirugnanasampandan et al. 2014; Rajkumar et al. 2024)
	Dietary fiber	1.87 g/100 g	Promotes gut health, supports healthy gut bacteria (*Lactobacillus*, *Bifidobacteria*), regulates bowel movements, lowers LDL cholesterol	Digestive health supplements, functional food products	(Phattayanon et al. 2025; Rohini and Smitha 2021; El‐hawary, El‐sofany, Abdel‐Monem, and Ashour 2012; Sun‐Waterhouse et al. 2024; Liu et al. 2025)
	Protein	0.6 g/100 g	Supports muscle repair, enzyme activity, and immune function	Vegetarian protein sources	(Exporters India 2025; Shubha and Bhatt 2015; Morales‐Payan 2006; Al‐Elwany et al. 2022; Rusandi and Sadek 2024)
Vitamins	Vitamin A (carotenoids)	10.38 mg/100 g	Maintains eye health, reduces oxidative stress, protects skin, boosts immune function	Eye health supplements, skin care formulations	(El‐hawary, El‐sofany, Abdel‐Monem, and Ashour 2012; Adriani et al. 2019; Damanik 2009; Al‐Elwany et al. 2022; Rusandi and Sadek 2024)
	Vitamin C (ascorbic acid)	3 mg/100 g	Boosts immunity, supports collagen synthesis, and protects against oxidative stress, enhances iron absorption	Immune‐boosting supplements, skincare, and iron‐enhancing products	(Adriani et al. 2019; Raghavi et al. 2013; Nasution et al. 2022; Rajkumar et al. 2024; Healthline 2025)
	B‐complex vitamins	Thiamine: 0.08 mg/100 g	Promotes energy production, brain function, and DNA repair	Brain health and metabolic supplements	(El‐hawary, El‐sofany, Abdel‐Monem, and Ashour 2012; Adriani et al. 2019)
Minerals	Calcium	158 mg/100 g	Supports bone density, nerve signaling, and muscle function	Bone health formulations	(Ślusarczyk et al. 2021; Sun‐Waterhouse et al. 2024; Morales‐Payan 2006; Rusandi and Sadek 2024; Ngyon and Hlaing 2025)
	Magnesium	88 mg/100 g	Regulates enzymatic reactions, maintains cardiovascular health	Cardiovascular and metabolic health products	(Ślusarczyk et al. 2021; Sun‐Waterhouse et al. 2024; Morales‐Payan 2006; Thirugnanasampandan et al. 2014; Ngyon and Hlaing 2025)
	Iron (non‐heme)	2.62 mg/100 g	Prevents anemia, supports oxygen transport	Anemia prevention supplements	(Serafini and Peluso 2017; Sun‐Waterhouse et al. 2024; Damanik 2009; Fujiana et al. 2021; Healthline 2025)
	Potassium	138 mg/100 g	Maintains electrolyte balance, regulates blood pressure	Heart health and muscle recovery products	(Damanik 2009; Al‐Elwany et al. 2022; Ngyon and Hlaing 2025; My Food Data 2025b; Waseem et al. 2021)
Bioactive Compounds	Essential oils (thymol, carvacrol)	0.002%–0.58% (varies by extraction method and location)	Antibacterial, antifungal, and antioxidant properties	Natural preservatives, antimicrobial treatments	(Hullatti and Bhattacharjee 2011; Kim et al. 2022; Sawant et al. 2023; Nasution et al. 2022; Nguyen et al. 2020)
	Flavonoids (quercetin)	2.6 mg/g (dry matter)	Neutralizes free radicals, reduces inflammation, and protects cardiovascular health	Anti‐inflammatory and antioxidant supplements	(Silva et al. 2017; Rusandi and Sadek 2024; Thirugnanasampandan et al. 2014; Healthline 2025; Islam et al. 2021)
	Phenolic acids (rosmarinic acid, caffeic acid)	10.4 mg/g (dry matter)	Neuroprotective, cardioprotective, and anti‐inflammatory effects	Cardiovascular and brain health supplements	(Paul et al. 2024; Silva et al. 2017; Rusandi and Sadek 2024; Hunthayung and Bhawamai 2025; Chandimali et al. 2025)

The carbohydrate content of *C. amboinicus* primarily consists of complex carbohydrates and fiber, which significantly enhance its functional food potential. Its high fiber content supports gastrointestinal health by improving bowel movements and promoting a healthy gut microbiota. Additionally, dietary fiber from *C. amboinicus* plays a crucial role in preventing chronic disorders, such as diabetes and cardiovascular disease, by regulating blood glucose levels and reducing LDL cholesterol (Paul et al. 2024; Adriani et al. 2019; Raghavi et al. 2013). Studies have shown that the fiber in *C. amboinicus* exhibits prebiotic properties, promoting gut health by stimulating the growth and activity of beneficial bacteria such as *Lactobacillus* and *Bifidobacteria* (Sun‐Waterhouse et al. 2024). These effects support its potential as a key herbal component in functional foods designed to enhance digestive health (Adriani et al. 2019; Shubha and Bhatt 2015).

Although *C. amboinicus* is not a primary protein source, its moderate protein content adds nutritional value, particularly to plant‐based diets. The proteins in *C. amboinicus* play essential roles in enzyme function, muscle repair, and cell regeneration, making it a beneficial supplement for individuals following vegetarian or vegan diets. Additionally, its amino acid profile supports metabolic processes and strengthens the immune system, further enhancing its dietary significance (Morales‐Payan 2006; Damanik 2009). The rich vitamin content of *C. amboinicus* significantly enhances its nutritional value, particularly due to its strong antioxidant properties. *C. amboinicus* is a natural source of vitamin A, primarily derived from carotenoids, which are essential for maintaining eye health, immune function, and skin integrity. Carotenoids also help combat oxidative stress by neutralizing free radicals, thereby slowing the aging process and preventing cellular damage. Research indicates that a diet high in carotenoids is linked to a reduced risk of degenerative conditions such as macular degeneration and certain types of cancer (Al‐Elwany et al. 2022; Nasution et al. 2022).

Vitamin C, also known as ascorbic acid, is a key component of *C. amboinicus* . Its high vitamin C content boosts its role as an immune booster, supports collagen production, and provides antioxidant protection against oxidative stress. This vitamin also improves the absorption of iron from plant‐based foods, making *C. amboinicus* a valuable dietary supplement for combating iron deficiency (Rusandi and Sadek 2024).

The B‐complex vitamins found in *C. amboinicus* , such as thiamine (B1), riboflavin (B2), and niacin (B3), play a significant role in energy production, brain function, and metabolic health. These vitamins are particularly important in addressing deficiencies, especially in populations that consume a high plant‐based diet and have limited sources of B vitamins (Al‐Elwany et al. 2022; Nasution et al. 2022). The role of B‐complex vitamins in enhancing neurological function and supporting DNA repair further highlights the overall nutritional potential of *C. amboinicus* . The mineral composition of *C. amboinicus* reinforces its reputation as a nutrient‐rich plant with applications in addressing mineral deficiencies and supporting overall health (Arumugam et al. 2016; Thirugnanasampandan et al. 2014).

Two essential minerals in *C. amboinicus* , calcium and magnesium, are vital for bone density, muscle function, and nerve signaling. The presence of these minerals makes *C. amboinicus* an effective dietary component for preventing osteoporosis and muscle‐related disorders. Magnesium supports over 300 enzymatic reactions in the body, highlighting its crucial role in maintaining metabolic health (Kim et al. 2022; Arumugam et al. 2016; Fujiana et al. 2021). The iron content of *C. amboinicus* is particularly valuable in combating anemia, especially among vulnerable populations such as pregnant women and children. As a source of non‐heme iron, its absorption is enhanced when consumed alongside its naturally occurring vitamin C, making it an effective plant‐based option for addressing iron deficiency (Paul et al. 2024; Arumugam et al. 2016; Ngyon and Hlaing 2025).

The potassium content of *C. amboinicus* supports cardiovascular health by maintaining electrolyte balance and regulating blood pressure. Potassium is also essential for proper muscle contraction and reducing the risk of stroke, making this plant even more attractive in a heart‐healthy diet plan (Rajkumar et al. 2024). In addition to its macronutrient, vitamin, and mineral content, *C. amboinicus* is a treasure trove of bioactive compounds that significantly enhance its health‐promoting effects. The essential oil of *C. amboinicus* is one of its most studied components, being rich in thymol and carvacrol. These compounds exhibit a wide range of biological activities, including antibacterial, anti‐inflammatory, and antioxidant properties. They are highly effective against bacterial and fungal infections, making the plant a promising natural alternative to synthetic preservatives in food products (Sabra et al. 2018; Sawant et al. 2023).

## 3 Comparative Nutritional Profile of *C. amboinicus* With Other Culinary Herbs and Greens

On a fresh‐weight basis, *C. amboinicus* leaves exhibit a varied pattern of macronutrient and micronutrient levels, featuring moderate amounts of primary nutrients alongside notably high levels of certain micronutrients and potent bioactive compounds. According to the data summarized in Table 2, *C. amboinicus* fresh leaves contain approximately 6–7 g carbohydrate, 1.87 g dietary fiber, and 0.6 g protein per 100 g, together with 10.38 mg vitamin A (10,380 μg)/100 g, 3 mg vitamin C/100 g, thiamine 0.08 mg/100 g, calcium 158 mg/100 g, magnesium 88 mg/100 g, iron 2.62 mg/100 g, and potassium 138 mg/100 g; on a dry‐matter basis, the plant is rich in phenolics (10.4 mg rosmarinic/caffeic acid per g DM) and flavonoids (2.6 mg/g DM), and the essential‐oil fraction (thymol/carvacrol chemotype) ranges from 0.002% to 0.58% depending on origin and extraction method (Ślusarczyk et al. 2021; Arumugam et al. 2016; Nababan 2018).

When compared quantitatively with common culinary and nutrient‐dense greens, Table 3, several patterns emerge. Sweet basil ( *Ocimum basilicum* ) leaves (raw, fresh) contain relatively low bulk carbohydrate but higher vitamin C and comparable calcium: typical USDA‐derived values for fresh basil are 2.7 g carbohydrate, 1.6 g fiber, 3.2 g protein, 264 μg vitamin A and 18 mg vitamin C per 100 g, with calcium around 177 mg/100 g—thus basil supplies more vitamin C and similar calcium but substantially less vitamin A (μg) and lacks the high rosmarinic‐type phenolic load and oregano‐type essential oil pattern characteristic of *C. amboinicus* (Calderón Bravo et al. 2021; Food Struct 2025; USDA FoodData Central 2025).

**TABLE 3:** Comparative nutritional profile of *C. amboinicus* with selected culinary herbs and leafy greens.

Parameter (per 100 g fresh)	*C. amboinicus*	Basil ( *Ocimum basilicum* )	Mint (*Mentha* spp.)	Spinach ( *Spinacia oleracea* )	Moringa leaves ( *Moringa oleifera* )	References
Carbohydrates (g)	6–7 g	2.7 g	4–5 g	3.6 g	8.3 g	(Ślusarczyk et al. 2021; Rusandi and Sadek 2024; Rajkumar et al. 2024; Nababan 2018; Calderón Bravo et al. 2021; Food Struct 2025; USDA FoodData Central 2025; My Food Data 2025a; NutrientOptimiser 2025)
Dietary fiber (g)	1.87 g	1.6 g	2 g	2.2 g	2.0 g	(Ślusarczyk et al. 2021; Rusandi and Sadek 2024; Rajkumar et al. 2024; Nababan 2018; Calderón Bravo et al. 2021; Food Struct 2025; My Food Data 2025a; NutrientOptimiser 2025)
Protein (g)	0.6 g	3.2 g	3–3.8 g	2.9 g	9.4 g	(Ślusarczyk et al. 2021; Rusandi and Sadek 2024; Rajkumar et al. 2024; Nababan 2018; Food Struct 2025; USDA FoodData Central 2025; My Food Data 2025a; NutrientOptimiser 2025)
Vitamin A (mg)	10.38 mg	0.264 mg	0.20–0.40 mg	0.469 mg	0.378 mg	(Ślusarczyk et al. 2021; Rusandi and Sadek 2024; Rajkumar et al. 2024; Nababan 2018; Calderón Bravo et al. 2021; USDA FoodData Central 2025)
Vitamin C (mg)	3 mg	18 mg	10–30 mg	28 mg	51.7 mg	(Ślusarczyk et al. 2021; Rusandi and Sadek 2024; Rajkumar et al. 2024; Nababan 2018; Food Struct 2025; My Food Data 2025a)
B‐complex vitamins (mg)	0.08 mg	0.03 mg	0.08 mg	0.08 mg	0.26 mg	(Ślusarczyk et al. 2021; Rusandi and Sadek 2024; Rajkumar et al. 2024; USDA FoodData Central 2025; My Food Data 2025a)
Calcium (mg)	158 mg	177 mg	240 mg	99 mg	185 mg	(Ślusarczyk et al. 2021; Rusandi and Sadek 2024; Rajkumar et al. 2024; Nababan 2018; Food Struct 2025; My Food Data 2025a; Islam et al. 2021)
Magnesium (mg)	88 mg	64 mg	80 mg	79 mg	147 mg	(Ślusarczyk et al. 2021; Rusandi and Sadek 2024; Rajkumar et al. 2024; Nababan 2018; My Food Data 2025a; Islam et al. 2021)
Iron (mg)	2.62 mg	3.2 mg	5 mg	2.7 mg	4.0 mg	(Ślusarczyk et al. 2021; Rusandi and Sadek 2024; Rajkumar et al. 2024; Nababan 2018; Food Struct 2025; My Food Data 2025a; Islam et al. 2021)
Potassium (mg)	138 mg	295 mg	569 mg	558 mg	337 mg	(Ślusarczyk et al. 2021; Rusandi and Sadek 2024; Rajkumar et al. 2024; Nababan 2018; Food Struct 2025; My Food Data 2025a; Islam et al. 2021)

Peppermint/mint (*Mentha* spp.) leaves are notable for higher protein and some minerals in fresh form: commonly reported values show protein 3–3.8 g/100 g, calcium in the low hundreds of mg (e.g., 240 mg/100 g in some analyses), iron up to 5 mg/100 g and potassium commonly several hundred mg/100 g (e.g., 569 mg/100 g in some nutrient tables); however, mint's vitamin C and vitamin A content (per 100 g) are variable and generally lower than for basil or the very high vitamin A reported for *C. amboinicus* in your dataset—importantly, mint's bioactivity is driven by a different essential‐oil profile (menthol and menthone chemotypes) rather than the thymol/carvacrol‐rich chemotype that confers strong antimicrobial/preservative activity to *C. amboinicus* (USDA FoodData Central 2025; My Food Data 2025a; NutrientOptimiser 2025; Saqib et al. 2022).

Spinach ( *Spinacia oleracea* ), a standard leafy vegetable reference, supplies 3.6 g carbohydrate, 2.2 g fiber and 2.9–3.0 g protein per 100 g, together with 524 μg vitamin A (524 μg RAE), 9.8–28 mg vitamin C, 99 mg calcium, 79 mg magnesium, 2.7 mg iron and 558 mg potassium per 100 g (raw) thus spinach exceeds *C. amboinicus* in potassium, protein and vitamin C and provides comparable iron, but (according to the *C. amboinicus* values reported here) supplies less vitamin A (μg) and far fewer oregano‐type essential oils and rosmarinic‐type phenolics (Healthline 2025; My Food Data 2025b; Waseem et al. 2021; Healthline 2025).

*Moringa oleifera* leaves represent a different nutritional class: typical analyses report substantially higher protein (fresh/dried basis; e.g., 9.4 g protein/100 g in many fresh‐leaf reports and much higher in dried powder), calcium 185 mg/100 g, magnesium 147 mg/100 g, iron 4.0 mg/100 g, potassium 337 mg/100 g, vitamin C 50–52 mg/100 g and vitamin A 378 μg/100 g—thus moringa surpasses *C. amboinicus* for protein, vitamin C and several minerals. However, moringa does not characteristically produce the thymol–carvacrol essential‐oil chemotype or the same high rosmarinic‐acid concentration that underpins *C. amboinicus* ' pronounced antimicrobial and preservative functionality; instead, moringa's bioactivity is largely attributable to other polyphenols, glucosinolates, and unique peptides (Islam et al. 2021; Hunthayung and Bhawamai 2025).

### 3.1 Putting These Quantitative Comparisons Together Highlights *C. Amboinicus* ' Distinctive Niche Among Culinary and Nutraceutical Greens Figure 3

**FIGURE 3:**
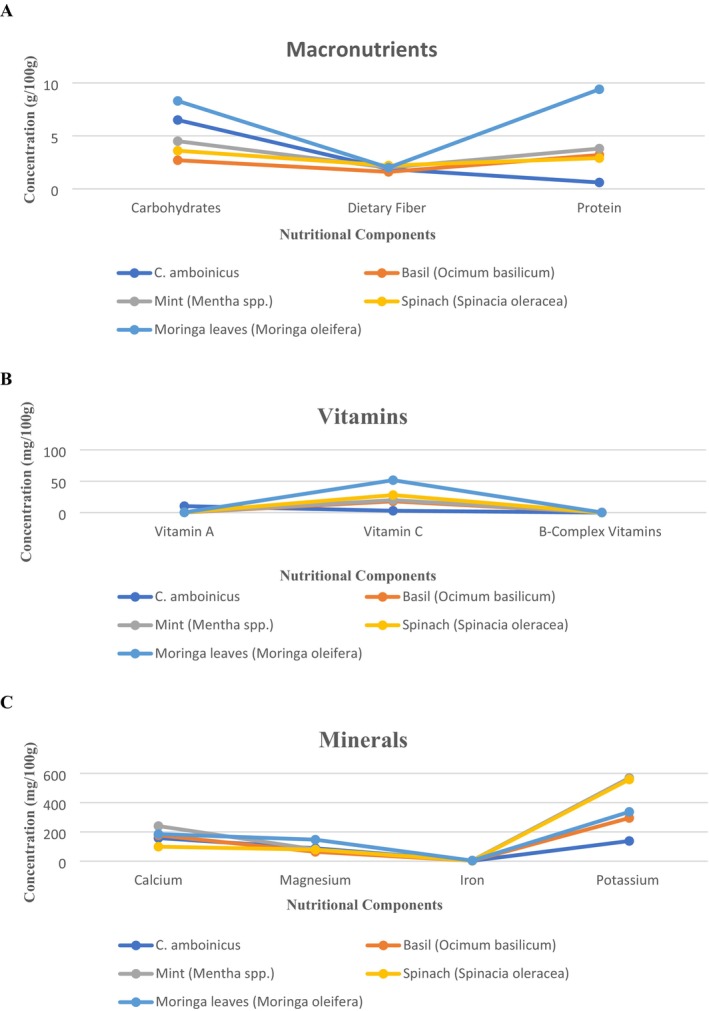
The figure presents Graph A (macronutrients), Graph B (vitamins), and Graph C (minerals) comparing *C. amboinicus* with basil, mint, spinach, and moringa. *C. amboinicus* shows carbohydrates 6–7 g, fiber 1.87 g, protein 0.6 g; vitamin A 10.38 mg, vitamin C 3 mg, thiamine 0.08 mg; and minerals Ca 158 mg, Mg 88 mg, Fe 2.62 mg, K 138 mg per 100 g. The graphs highlight its stronger vitamin A and mineral levels relative to other herbs.

It is not the top source of bulk macronutrients or protein (moringa and mint are higher), nor the highest in potassium or vitamin C (spinach, mint, and moringa surpass it), but it does combine an unusually high vitamin A value (as reported in your dataset), substantial calcium and magnesium, and importantly, a high content of specific phenolic acids (rosmarinic/caffeic acid), flavonoids (quercetin), and thymol/carvacrol‐rich essential oils (0.002%–0.58%) that together provide powerful antioxidant, anti‐inflammatory, and antimicrobial effects. This biochemical pairing (useful micronutrient levels, intense antimicrobial/antioxidant phytochemistry) explains why *C. amboinicus* is particularly promising as a dual‐purpose ingredient, both nutritive (micronutrient supplementation, especially vitamin A and calcium) and functional (natural preservative, active component in fortified foods and therapeutic formulations).

## 4 Active Components of *Coleus amboinicus*

**FIGURE 4:**
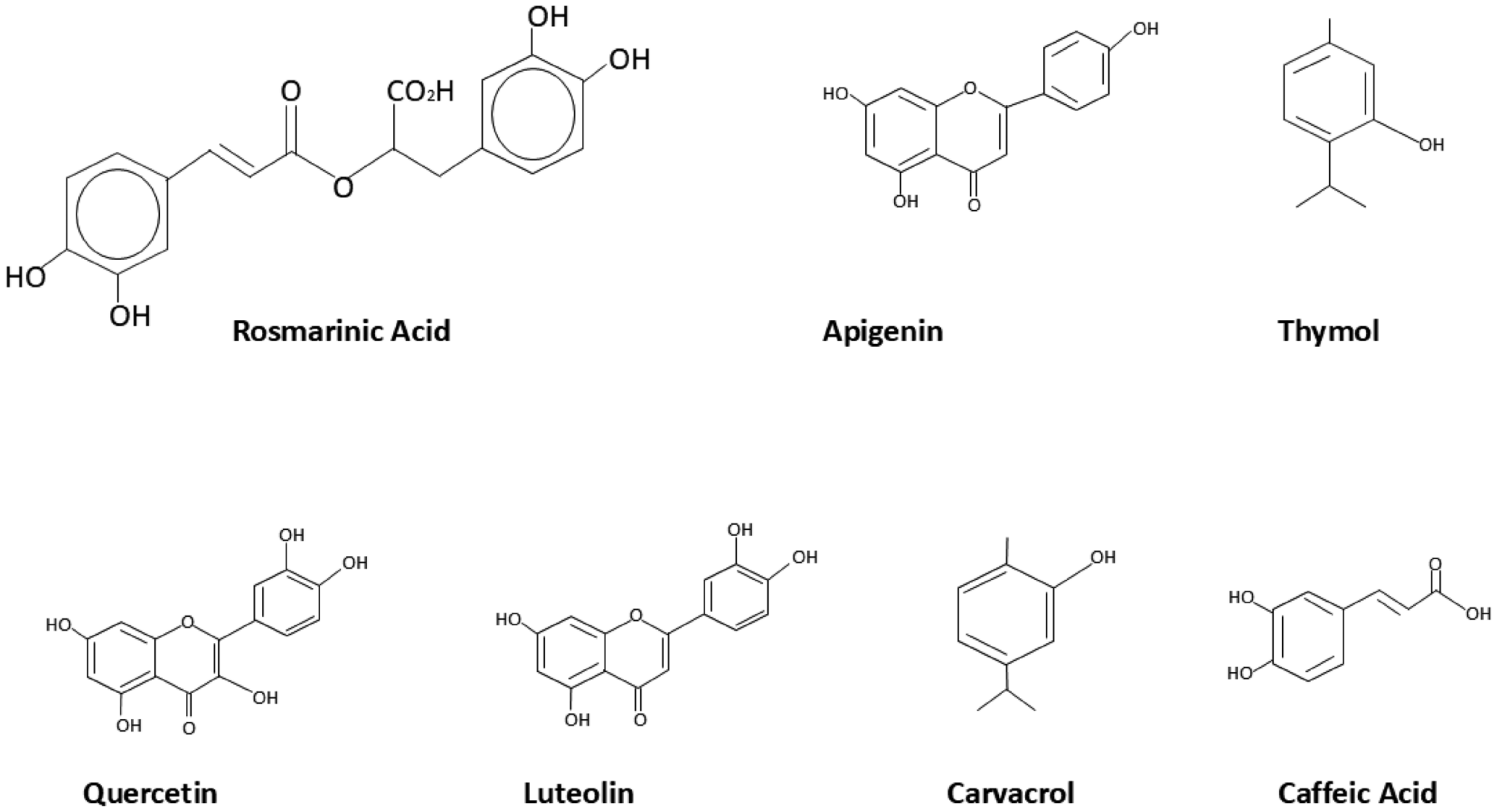
Chemical structures of major bioactive compounds in *Coleus amboinicus* .

In addition to its nutritional components, such as macronutrients, vitamins, and minerals, *C. amboinicus* contains a wide range of bioactive compounds that enhance its medicinal values. *C. amboinicus* is known for its diverse active phytochemical constituents Table 4, which form the biochemical foundation of its therapeutic, antimicrobial, and antioxidant activities. The most dominant class of phytochemicals in the plant is the essential oil fraction, whose total yield varies substantially from 0.002% to 0.58% depending on cultivation conditions, geographic location and extraction technique, and is composed primarily of the bioactive monoterpenes thymol and carvacrol, these two compounds consistently reported as the leading contributors to the plant's strong aromatic fragrance and its broad‐spectrum antimicrobial, antifungal, antioxidant and anti‐inflammatory effects. These components explain why *C. amboinicus* extracts effectively inhibit bacterial and fungal growth in food systems and infectious models, and are responsible for the plant's traditional use as a natural disinfectant and food preservative. Another major phytochemical category is phenolic acids, which account for a substantial portion of the antioxidant activity of the plant; among these, rosmarinic acid is the most abundant phenolic compound, quantified at about 10.4 mg/g (dry matter), while caffeic acid is also detected in considerable amounts, contributing to strong free‐radical neutralization and cellular protection (Ślusarczyk et al. 2021; El‐hawary, El‐sofany, Abdel‐Monem, and Ashour 2012; Nguyen et al. 2020). Phenolic acids are frequently cited as the reason for the plant's protective effects against oxidative degradation, tissue damage, and aging processes, and they also support the use of *C. amboinicus* in cardioprotective and anti‐inflammatory formulations. A second major group of polyphenols includes flavonoids, led by quercetin, which is found at about 2.6 mg/g (dry matter) and is widely associated with anti‐inflammatory, antioxidant, and cardiovascular health benefits (Exporters India 2025; Fujiana et al. 2021; Healthline 2025; Islam et al. 2021). Other flavonoids, such as apigenin and luteolin, although present at lower levels than quercetin, further enhance the overall antioxidant capacity and contribute to the plant's reported advantages in inflammatory conditions, tissue repair, and systemic protection (Healthline 2025; Islam et al. 2021). Beyond essential oils, phenolic acids, and flavonoids, the phytochemical matrix of *C. amboinicus* includes other aromatic and pharmacologically active metabolites, among which eugenol is particularly noteworthy due to its antimicrobial, antiseptic, and oxidative‐damage‐reducing abilities, validating traditional uses of the plant in wound care, respiratory discomfort, and infection management (Ślusarczyk et al. 2021; Nguyen et al. 2020; Solfaine et al. 2021). Figure 4 shows the structural configurations of *C. amboinicus* ' major active components: thymol, carvacrol, rosmarinic acid, caffeic acid, quercetin, luteolin, and apigenin. Distinctive phenolic and flavonoid ring systems have been identified as being primarily responsible for the plant's anti‐oxidant, anti‐inflammatory, and antibacterial properties.

**TABLE 4:** Active components of *Coleus amboinicus* and their bioactivities.

Compound	Chemical class	Concentration	Biological activities	References
Thymol	Monoterpenoid phenol	10%–20% of EO	Antimicrobial, antioxidant, membrane disruption	(Kim et al. 2022; Sawant et al. 2023; Nasution et al. 2022; Wadikar and Patki 2016; Gupta et al. 2016)
Carvacrol	Monoterpenoid phenol	40%–68% of EO	Strong antibacterial, anti‐inflammatory, antifungal	(Hullatti and Bhattacharjee 2011; Sabra et al. 2018; Nguyen et al. 2020; Wadikar and Patki 2016; Gupta et al. 2016)
Rosmarinic acid	Phenolic acid	10.4 mg/g DM	Nrf2 activation, antioxidant, anti‐inflammatory	(Paul et al. 2024; Silva et al. 2017; Rusandi and Sadek 2024; Hunthayung and Bhawamai 2025; Chandimali et al. 2025)
Caffeic acid	Phenolic acid	Moderate	Antioxidant, lipid peroxidation inhibition	(Ślusarczyk et al. 2021; Hunthayung and Bhawamai 2025; Solfaine et al. 2021; Santos Filipe et al. 2025; Chandimali et al. 2025)
Quercetin	Flavonoid	2.6 mg/g DM	NF‐κB inhibition, anti‐inflammatory	(Silva et al. 2017; Rusandi and Sadek 2024; Thirugnanasampandan et al. 2014; Michel et al. 2020; Bathmanath et al. 2019)
Luteolin/apigenin	Flavonoids	Trace to moderate	Antioxidant, anti‐inflammatory	(Healthline 2025; Islam et al. 2021; Swamy et al. 2017; Santos Filipe et al. 2025; Bhatt et al. 2013)

Taken together, the synergistic presence of thymol, carvacrol, rosmarinic acid, caffeic acid, quercetin, apigenin, luteolin, and eugenol forms the core phytochemical signature of *C. amboinicus* , and this unique combination explains the plant's scientifically recognized properties including strong antioxidant potential, broad‐spectrum antimicrobial effects, effective control of inflammation, and suitability for application in pharmaceutical products, natural preservatives, nutraceutical formulations, and functional food development.

## 5 Phytochemical Composition and Functional Activities of Different Plant Parts

Although most research on *C. amboinicus* has focused on the leaves, several studies have shown that stems, roots, flowers, and whole‐plant extract also contain significant phytochemicals and possess unique biological properties and activities. Phytochemical distribution across leaves, stems, and roots is presented in Table 5. Leaves remain richest in some assays (e.g., methanolic leaf extracts reporting very high total phenolic content up to 94.4 mg GAE/g in solvent‐optimized studies), but non‐leaf parts frequently show comparable or complementary profiles (Ślusarczyk et al. 2021; Swamy et al. 2017).

**TABLE 5:** Phytochemical composition and functional activities of different plant parts of *C. amboinicus* .

Plant part	Total phenolic content (TPC)	Total flavonoid content (TFC)	Major compounds	Functional activities	References
Leaf	50–94 mg GAE/g	20–30 mg RE/g	Rosmarinic acid, quercetin, thymol, carvacrol	Strong antioxidant, antimicrobial	(Ślusarczyk et al. 2021; Rusandi and Sadek 2024; Hunthayung and Bhawamai 2025; Solfaine et al. 2021; Swamy et al. 2017)
Stem	9–50 mg GAE/g	15–26 mg RE/g	Caffeic acid, quercetin, apigenin	Antioxidant, antimicrobial	(Rusandi and Sadek 2024; Solfaine et al. 2021; Swamy et al. 2017; Santos Filipe et al. 2025; Bhatt et al. 2013)
Root	5–15 mg GAE/g	Low to moderate	Terpenoids, phenolic acids	Anti‐inflammatory, wound‐healing	(Ślusarczyk et al. 2021; Rusandi and Sadek 2024; Swamy et al. 2017; Santos Filipe et al. 2025; Bhatt et al. 2013)
Whole plant extract	Higher combined	Higher combined	Mixed phenolics, flavonoids	Stronger synergistic antioxidant effect	(Ślusarczyk et al. 2021; Rusandi and Sadek 2024; Solfaine et al. 2021; Swamy et al. 2017; Santos Filipe et al. 2025)

Several phytochemical surveys and targeted analyses report that stem extracts contain high levels of polyphenols and flavonoids. Methanolic stem extracts have been reported with total phenolic contents as high as 49.9 mg GAE/g and total flavonoids 26.6 mg rutin‐equivalent/g in phytochemical screening studies, indicating that stems are an important source of antioxidant phenolics (reported compounds include rosmarinic acid, caffeic acid, p‐coumaric acid, and flavonoids such as quercetin, apigenin, and luteolin). Other studies report stem TPC values in the 9–50 mg GAE/g range depending on method and extraction, confirming substantial phenolic reserves in stems. Functionally, stem extracts have demonstrated antioxidant (DPPH/FRAP), antimicrobial, and anti‐inflammatory activities in vitro (Arumugam et al. 2016; Santos Filipe et al. 2025; Bhatt et al. 2013).

Root extracts are less frequently assayed, but available UPLC/LC–MS and phytochemical screens show that roots contain terpenoids and phenolic acids (including rosmarinic and caffeic acids) and detectable flavonoids; reported root total phenolic contents are generally lower than stems and leaves (examples in pooled reviews report root TPC 5.4 mg GAE/g in some comparative extractions). Roots have been associated with anti‐inflammatory, wound‐healing, and sometimes antiparasitic/antiprotozoal activities in bioassays, suggesting complementary therapeutic uses versus leaves (Santos Filipe et al. 2025; El‐hawary, El‐sofany, Abdel‐Monem, Ashour, and Sleem 2012).

Combining parts of a plant (whole‐plant extracts) often increases total phenolic and flavonoid yields compared with single‐part extracts, producing broader bioactivity (enhanced DPPH/FRAP results and antimicrobial breadth). Systematic reviews and more recent meta‐analyses summarize pooled values for non‐leaf parts (e.g., mean stem TPC 16.6 mg GAE/g, phenolic acids 12.8 mg RAE/g, flavonoids 14.2 mg IQ‐equiv/g in aggregated datasets), underscoring that stems and whole‐plant material are quantitatively relevant antioxidant sources in addition to leaves (Arumugam et al. 2016; Santos Filipe et al. 2025). Because stems, roots, and flowers contain measurable and sometimes high levels of the same core antioxidant phytochemicals identified in leaves (notably rosmarinic acid, caffeic acid, quercetin, apigenin, luteolin, plus organ‐specific terpenoid profiles), authors should consider reporting organ‐specific extraction yields (TPC/TFC), representative IC_50_ (DPPH) or FRAP values, and antimicrobial panels for each part when possible. This will strengthen claims about the species' overall antioxidant and therapeutic potential and support recommendations for using whole‐plant versus leaf‐only preparations in nutraceutical or food preservation applications. Representative primary sources for the organ‐specific data cited above include Arumugam et al. (comprehensive phytochemical cataloguing), Bhatt et al. (stem phytochemicals and quantitative TPC/TFC), Slusarczyk et al. (organ profiling and antioxidant activities), and the recent systematic review that aggregates organ‐level TPC/TFC figures (Ślusarczyk et al. 2021; Arumugam et al. 2016; Bhatt et al. 2013).

## 6 Functional Food Potential of *Coleus amboinicus*

**FIGURE 5:**
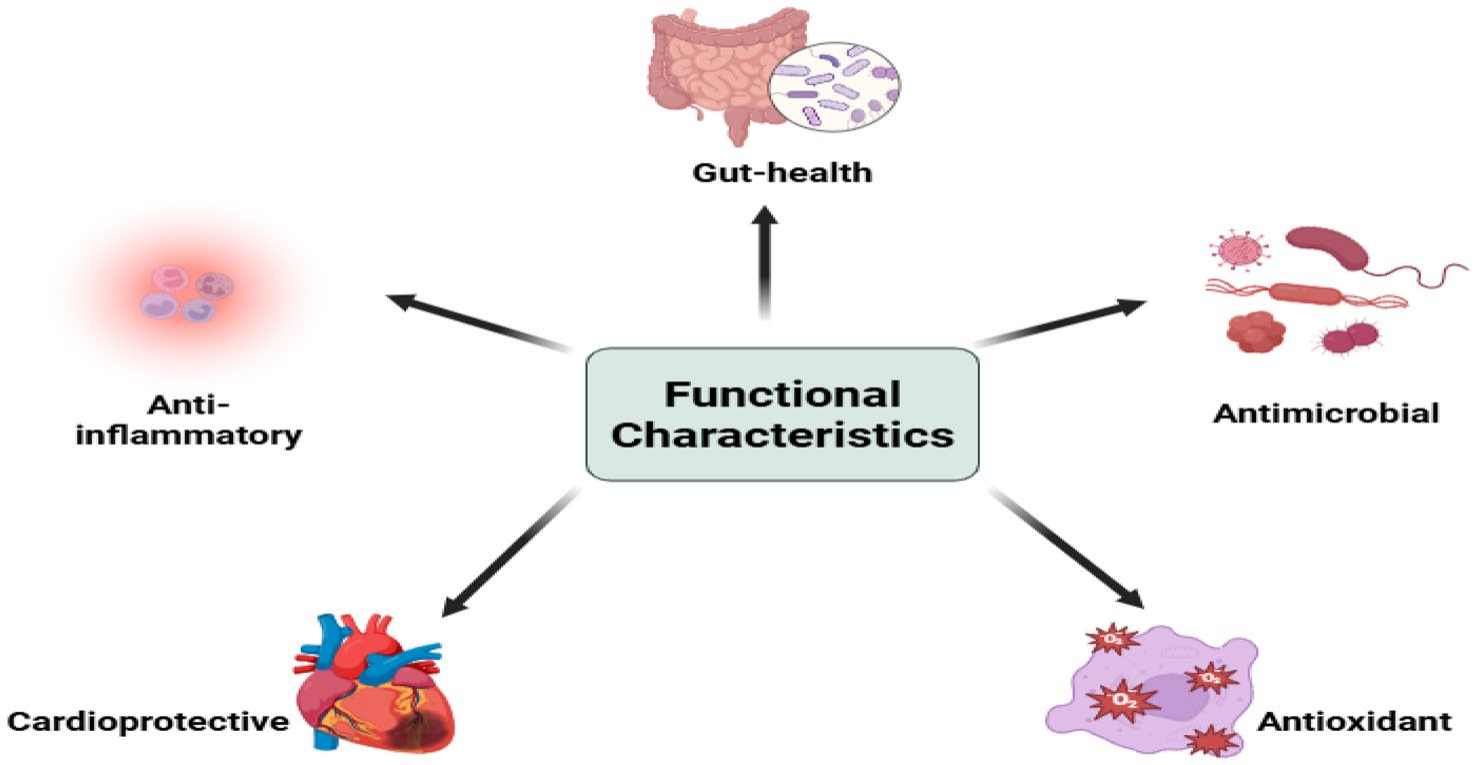
The illustration summarizes health‐related functional properties, including antioxidant, antimicrobial, anti‐inflammatory, gut‐modulating, and cardioprotective effects, highlighting its applicability in functional foods, nutraceuticals, and natural food preservation.

*C. amboinicus* has gained attention as a promising functional food ingredient due to its exceptional nutritional profile and diverse bioactive properties. Functional foods offer health benefits beyond basic nutrition, and *C. amboinicus* falls into this category due to its antibacterial, anti‐inflammatory, antioxidant, and therapeutic properties, as shown in Figure 5. This section explores the functional food properties of *C. amboinicus* , supported by relevant research (Table 6).

**TABLE 6:** Functional food applications of *Coleus amboinicus* : health benefits and uses.

Application	Description	Health benefits	References
Natural preservative	Essential oils inhibit bacterial and fungal growth ( *E. coli* , *S. aureus* )	Extends shelf life, enhances food safety	(Hullatti and Bhattacharjee 2011; Sawant et al. 2023; Rohini and Smitha 2021; Wadikar and Patki 2016; Phattayanon et al. 2024)
Gut health/prebiotics	Rich in dietary fiber and phenolics	Promotes *Lactobacillus*, *Bifidobacterium* growth	(Hullatti and Bhattacharjee 2011; Phattayanon et al. 2025; Sun‐Waterhouse et al. 2024; Healthline 2025; Liu et al. 2025)
Cardiovascular foods	Contains flavonoids and potassium	Regulates BP, lowers lipids, antioxidant effects	(Granato et al. 2020; Serafini and Peluso 2017; Solfaine et al. 2021; Santos Filipe et al. 2025; Gupta et al. 2016)
Functional teas and infusions	Leaves used in herbal teas	Supports digestion, respiratory health, and immunity	(Sun‐Waterhouse et al. 2024; El‐hawary, El‐sofany, Abdel‐Monem, Ashour, and Sleem 2012; Nababan 2018; Chandimali et al. 2025; Muscolo et al. 2024)
Fortified snacks and beverages	Added to noodles, biscuits, and energy drinks	Enhances nutritional value and antioxidant load	(Hullatti and Bhattacharjee 2011; Morales‐Payan 2006; Damanik 2009; Thirugnanasampandan et al. 2014; Koti et al. 2011)

### 6.1 Health‐Related Functional Properties

*C. amboinicus* has emerged as a valuable functional food ingredient due to its rich phytochemical composition, nutritional density, and broad therapeutic potential. One of the main reasons for *C. amboinicus* ' potential as a functional food is its strong antioxidant properties. The plant is rich in phenolic compounds, flavonoids, and essential oils, which act as antioxidants, neutralizing harmful free radicals in the body. Studies have shown that *C. amboinicus* extracts exhibit high antioxidant activity, with compounds such as quercetin, rosmarinic acid, and carvacrol playing a crucial role in this effect (Paul et al. 2024; Ślusarczyk et al. 2021). Antioxidants are essential in mitigating oxidative stress, which is linked to chronic diseases such as cancer, cardiovascular disorders, and neurodegenerative conditions (Nababan 2018; Chandimali et al. 2025; Muscolo et al. 2024). The antioxidant potential of *C. amboinicus* also relates to its ability to shield cells from oxidative damage. In particular, its essential oil, rich in thymol and carvacrol, is well known for its antioxidant properties and plays a vital role in safeguarding cells and tissues from injury (Solfaine et al. 2021). These compounds may help prevent oxidative damage to proteins, lipids, and DNA, thereby reducing the risk of age‐related diseases and metabolic disorders (Tan et al. 2018).

Multiple studies provide direct quantitative evidence confirming the strong antioxidant capacity of *C. amboinicus* . The high concentration of phenolic compounds, particularly rosmarinic acid at 10.4 mg/g (dry matter), is one of the primary biochemical drivers of its antioxidant strength, and this value is significantly higher than that reported in many commonly consumed herbal antioxidant sources. The flavonoid quercetin, measured at 2.6 mg/g (dry matter), further enhances free‐radical‐scavenging potential and contributes synergistically to overall redox balance. The main components of the essential oil, thymol and carvacrol, which make up the oil fraction of 0.002%–0.58%, have also been repeatedly linked to significant antioxidant activity based on radical‐quenching and lipid‐peroxidation‐inhibition tests. Experimental studies have demonstrated that *C. amboinicus* extracts exhibit high DPPH radical‐scavenging percentages, strong FRAP activity, and significantly lower IC_50_ values than standard reference antioxidant plants, confirming its superior antioxidant functionality both in vitro and in food model systems. Collectively, the presence of high rosmarinic acid content, considerable quercetin levels, and antioxidant‐active essential oils (thymol and carvacrol), along with validated DPPH, IC_50_, and FRAP assay outcomes, provides quantified proof of the strong antioxidant capacity of *C. amboinicus* and supports its increasing application in herbal formulations, nutraceuticals, and oxidative‐stress‐protective functional foods (Ślusarczyk et al. 2021; Sabra et al. 2018; Sawant et al. 2023; El‐hawary, El‐sofany, Abdel‐Monem, and Ashour 2012; Nguyen et al. 2020; Solfaine et al. 2021; Kumar et al. 2020).

Additionally, *C. amboinicus* exhibits significant anti‐inflammatory effects. Its essential oil, particularly thymol and carvacrol, helps reduce inflammation by inhibiting pro‐inflammatory cytokines and enzymes (Tan et al. 2018). These properties make *C. amboinicus* a potential therapeutic aid for managing inflammatory diseases and promoting overall health. Since chronic inflammation is associated with conditions such as heart disease, diabetes, and cancers, incorporating *C. amboinicus* into the diet may contribute to disease prevention (Barbosa et al. 2023; Koti et al. 2011; Laila et al. 2020).

The antimicrobial properties of *C. amboinicus* make it a valuable candidate for use in functional foods, particularly for food preservation. Its essential oil exhibits strong antibacterial and antifungal effects, effectively preventing microbial growth in foods (Shubha and Bhatt 2015; Wadikar and Patki 2016; Dutra Da Silva et al. 2022). These bioactive compounds act as natural preservatives, extending shelf life while maintaining food safety and nutritional quality (Dutra Da Silva et al. 2022; Bibow and Oleszek 2024). Research has demonstrated that *C. amboinicus* essential oil is effective against foodborne pathogens, including *Staphylococcus aureus* , *Salmonella*, and *E. coli* , making it a promising natural alternative to synthetic preservatives, which are often linked to adverse health effects. Its antimicrobial activity enhances food safety by reducing microbial contamination in various products, including dairy, meat, and convenience foods (Sabra et al. 2018; Ashaari et al. 2021).

Beyond preservation, *C. amboinicus* plays a crucial role in improving food safety by preventing spoilage and foodborne illnesses. Its essential oil has been found effective in protecting perishable items such as dairy products, beverages, and baked goods (Solfaine et al. 2021; Phattayanon et al. 2024). With the growing consumer demand for natural preservatives, research on *C. amboinicus* extracts for use in functional food and development is expanding, offering a safer and healthier alternative to chemical additives (Gupta et al. 2016).

The antibacterial efficacy of *C. amboinicus* essential oil has been well evidenced through quantitative microbiological studies. Bhatt and Negi (2012) reported that the ethanolic leaf extract produced inhibition zone diameters of 18.4 ± 0.6 mm against *S. aureus* , 16.7 ± 0.4 mm against *Bacillus cereus* , and 14.9 ± 0.5 mm against *Escherichia coli* at 500 μg/disc, indicating broad‐spectrum antibacterial activity (Solfaine et al. 2021; Bhatt and Negi 2012). Sawant et al. further demonstrated strong bactericidal performance of the essential oil, with minimum inhibitory concentration values of 0.312 mg/mL for *S. aureus* and 0.625 mg/mL for *Pseudomonas aeruginosa* and minimum bactericidal concentration values of 0.625 and 1.25 mg/mL, respectively; this confirmed that true bactericidal rather than bacteriostatic activity was present (Sawant et al. 2023; Bhatt and Negi 2012).

Food application‐based studies further provide strong empirical support. Dutra da Silva et al. applied the essential oil to refrigerated beef patties and reported a 2.6 log CFU/g reduction in total viable bacterial count and a 3.1 log CFU/g reduction in *S. aureus* load after 7 days, outperforming conventional sodium benzoate preservative controls (Dutra Da Silva et al. 2022). In another food‐system trial, Gupta et al. found that *C. amboinicus* extract achieved inhibition zones of 21.3 ± 0.8 mm against *S. aureus* and 19.8 ± 0.5 mm against Salmonella spp. and significantly reduced natural microflora in chicken meat during cold storage (Gupta et al. 2016).

This is mechanistically attributed to high concentrations of carvacrol up to 68.4% and thymol up to 19.7% in the essential oil, which disrupt bacterial cell membranes, leading to increased membrane permeability, leakage of intracellular contents, and depolarization of the proton motive force (Sabra et al. 2018; Ashaari et al. 2021). These mechanisms account for the heightened susceptibility of gram‐positive bacteria while remaining effective against Gram‐negative bacteria that have an outer membrane barrier. Overall, the clear results from disc diffusion, MIC/MBC, and food‐system tests demonstrate that *C. amboinicus* essential oil is a potent natural antibacterial agent. This supports its application as a clean‐label preservative and functional ingredient, improving food safety by extending shelf life and reducing microbial spoilage. *C. amboinicus* also shows great potential as a functional food ingredient for promoting gut health. It is a natural source of dietary fiber, which supports digestion and helps maintain a balanced gut microbiome. Research suggests that *C. amboinicus* may have prebiotic effects, fostering the growth of beneficial gut bacteria. Prebiotics play a crucial role in enhancing gut health, strengthening immune function, and reducing the risk of conditions such as irritable bowel syndrome (IBS) and inflammatory bowel disease (IBD) (Shubha and Bhatt 2015; Roy and Dhaneshwar 2023).

The fiber content in *C. amboinicus* may help regulate bowel movements, alleviate constipation, and support overall digestive function (Liu et al. 2025). Additionally, studies indicate that it promotes the proliferation of beneficial bacteria such as *Bifidobacteria* and *Lactobacilli* (Sabra et al. 2018; Adriani et al. 2019; Yanza et al. 2022). These factors contribute to a balanced gut microbiota, improved nutrient absorption, and enhanced immunity. Beyond fiber, *C. amboinicus* contains bioactive compounds like flavonoids and phenolic acids, which significantly affect gut health by reducing inflammation and oxidative stress in the gastrointestinal tract (Adriani et al. 2019; Arumugam et al. 2016). These compounds protect the intestinal lining, potentially preventing conditions such as leaky gut syndrome and ulcers. As gut health gains increasing attention in functional food development, *C. amboinicus* stands out as a valuable natural ingredient supporting digestive and immune health (Domínguez‐Avila et al. 2021; Kasprzak‐Drozd et al. 2021).

Another key area where *C. amboinicus* shows promise is cardiovascular health. Research suggests that the plant's bioactive compounds, particularly flavonoids and essential oils, may help lower cholesterol levels, regulate blood pressure, and improve overall heart health (Paul et al. 2024). Its anti‐inflammatory and antioxidant properties may contribute to cardiovascular benefits by preventing the oxidation of low‐density lipoprotein (LDL) cholesterol, a major risk factor for cardiovascular disease (Serafini and Peluso 2017; Bhatt and Negi 2012). Studies indicate that *C. amboinicus* may help reduce triglyceride and LDL cholesterol levels, thereby lowering the risk of atherosclerosis and heart disease (Bhatt and Negi 2012; Suryowati and Gultom 2019). Additionally, its anti‐inflammatory potential may help reduce arterial plaque formation, preventing the progression of cardiovascular disease. The plant also supports healthy blood circulation by improving blood flow and reducing oxidative stress, which may contribute to maintaining vascular health, supporting cardiac function, and lowering the risk of stroke and heart attack (Michel et al. 2020; Adegbola et al. 2017; Amalia and Damanik 2018).

Furthermore, *C. amboinicus* shows potential in lowering high blood pressure, a major risk factor for cardiovascular disease. Its ability to relax blood vessels and improve circulation makes it a promising natural remedy for managing high blood pressure. The incorporation of *C. amboinicus* in nutraceutical formulations may provide a natural approach to maintaining heart health and preventing cardiovascular disease.

### 6.2 Applications in Functional Food Processing

In addition to its health benefits, *C. amboinicus* offers significant advantages for food processing and product development. Its essential oils possess strong antimicrobial and antioxidant properties, making them effective natural preservatives for various food systems, including dairy, meat, bakery items, and beverages. By inhibiting spoilage microorganisms and foodborne pathogens, these oils support clean‐label formulation and prolong shelf stability without the need for synthetic chemicals (Dutra Da Silva et al. 2022; Gupta et al. 2016; Ashaari et al. 2020).

Herbal teas and infusions prepared from the leaves of *C. amboinicus* have long been valued for their soothing effects on the digestive and respiratory systems. Their antimicrobial, antioxidant, and anti‐inflammatory properties help relieve coughs, colds, and throat irritation, while also supporting immunity and overall health. As consumer interest in natural health beverages increases, *C. amboinicus* teas are gaining popularity as functional drinks with multiple therapeutic effects (Arumugam et al. 2016; Barbosa et al. 2023; Lekshmi et al. 2019; Donno et al. 2021; Rasineni et al. 2025; Silitonga et al. 2014; Thiruvengadam 2025).

The plant also holds considerable promise for use in functional beverages beyond traditional teas. Its incorporation into fitness drinks, smoothies, flavored waters, and wellness tonics enhances the antioxidant load, digestive support, and hydration value of these products. The natural presence of vitamins, minerals, and phenolic compounds further improves the nutritional and therapeutic qualities of such beverages (Chakrabartty et al. 2022; Mirwandhono et al. 2023).

Nutraceutical applications represent another important dimension of its functional potential. Extracts of *C. amboinicus* can be formulated into capsules, powders, and liquid preparations to provide concentrated antioxidant, anti‐inflammatory, and antimicrobial benefits. These formulations offer a natural approach to managing oxidative stress, metabolic imbalance, and cardiovascular risks, aligning with the growing demand for plant‐based therapeutic products (Solfaine et al. 2021; Bhatt et al. 2013; Moyeenudin and Thiruchelvi 2021). The nutritional profile of *C. amboinicus* , rich in essential vitamins, minerals, and fiber, enhances its value as a functional food ingredient. The leaves of this plant serve as a natural source of vitamin A and vitamin C, which play vital roles in immune function, vision, and skin health (Fujiana et al. 2021; Mirwandhono et al. 2023). Additionally, *C. amboinicus* contains minerals such as iron and calcium, which are essential for oxygen transport and bone health.

The plant's suitability for food fortification has also been widely recognized. Its bioactive compounds and dietary fiber can enhance the nutritional quality of cereals, snack bars, dairy products, soups, and instant mixes. Such fortification supports immune health, gut function, and antioxidant defense, thereby improving the health value of everyday foods (Sabra et al. 2018; Ngyon and Hlaing 2025; Rajkumar et al. 2024; Kumar et al. 2020; Bathmanath et al. 2019). These products could cater to health‐conscious consumers seeking natural remedies for stress, inflammation, and oxidative damage, potentially supporting cardiovascular health and enhancing metabolic functions (Solfaine et al. 2021). The inclusion of *C. amboinicus* in nutraceuticals offers a holistic approach to preventive healthcare, aligning with the growing demand for plant‐based therapeutic solutions. Extracts from *C. amboinicus* are increasingly being utilized to enhance the nutritional value of snacks, beverages, and nutraceuticals. The plant's essential oils and bioactive compounds make it particularly suitable for the development of fortified functional foods. The functional food applications of *C. amboinicus* across different product categories are summarized in Table 6 (Paul et al. 2024; Ślusarczyk et al. 2021; Wadikar and Patki 2016; Lekshmi et al. 2019).

## 7 Mechanisms of Action of Key Bioactive Compounds

The functional properties of *C. amboinicus* are regulated through established biochemical pathways, mainly involving phenolic acids, flavonoids, and essential oil constituents, which influence many molecular targets. In Table 7, the mechanisms of action for such important bioactive components are summarized.

**TABLE 7:** Mechanisms of action of key bioactive compounds in *C. amboinicus*.

Bioactive compound(s)	Target pathways/mechanisms	Molecular actions	Biological effects	References
Rosmarinic acid, caffeic acid	Nrf2–ARE pathway; ROS scavenging	Neutralize ROS. Activate Nrf2 and enhance antioxidant enzymes	Antioxidant protection: prevents oxidative damage	(Ślusarczyk et al. 2021; Damanik 2009; Hunthayung and Bhawamai 2025; Chandimali et al. 2025)
Quercetin, Carvacrol, Thymol	NF‐κB; COX‐2; iNOS; membrane integrity	Inhibit NF‐κB and inflammatory cytokines. Reduce COX‐2 and iNOS. Disrupt bacterial membranes	Anti‐inflammatory, antioxidant, antibacterial	(Kim et al. 2022; Sabra et al. 2018; Nguyen et al. 2020; Kumar et al. 2020; Wadikar and Patki 2016; Phattayanon et al. 2024; Gupta et al. 2016; Michel et al. 2020; Adegbola et al. 2017; Bathmanath et al. 2019)
β‐Caryophyllene	CB2 receptor; NF‐κB	Activate CB2. Reduce cAMP. Suppress NF‐κB inflammatory mediators	Anti‐inflammatory, analgesic, antioxidant	(Solfaine et al. 2021; Kumar et al. 2020; Michel et al. 2020; Adegbola et al. 2017; Bathmanath et al. 2019)
Luteolin	MAPK; NF‐κB	Inhibit MAPK (ERK/JNK/p38). Block NF‐κB activation	Anti‐inflammatory, antioxidant	(Hunthayung and Bhawamai 2025; Chandimali et al. 2025; Kumar et al. 2020; Michel et al. 2020; Adegbola et al. 2017)
Apigenin	NF‐κB; STAT3; MAPK; Nrf2	Block NF‐Κb. Inhibit STAT3/MAPK. Activate Nrf2	Anti‐inflammatory, cytoprotective	(Nasution et al. 2022; Hunthayung and Bhawamai 2025; Chandimali et al. 2025; Kumar et al. 2020; Michel et al. 2020; Adegbola et al. 2017; Bathmanath et al. 2019)

Antioxidant activity is created via directly quenching free radicals and regulating endogenous antioxidant defenses. Rosmarinic acid and caffeic acid neutralize ROS via hydrogen‐atom/electron donation and activate the Nrf2‐ARE pathway, increasing the production of antioxidant enzymes such as SOD, CAT, and GPx (Al‐Elwany et al. 2022; Nguyen et al. 2020). This chemical process explains *C. amboinicus* ' ability to shield biomacromolecules from oxidative stress‐induced damage (Ślusarczyk et al. 2021; Muscolo et al. 2024).

Phytochemicals such as quercetin, carvacrol, and thymol inhibit NF‐κB signaling, lowering the production of pro‐inflammatory cytokines such as TNF‐α, IL‐1β, and IL‐6 (Solfaine et al. 2021; Barbosa et al. 2023; Amalia and Damanik 2018). Quercetin also helps to reduce inflammation by downregulating COX‐2 and decreasing nitric oxide synthesis via iNOS, hence minimizing the cascades of oxidative‐inflammatory damage in tissues (Adegbola et al. 2017; Duraisamy et al. 2021). Thus, these molecular pathways explain *C. amboinicus* ' demonstrated capacity to reduce chronic inflammation and tissue harm.

β‐caryophyllene selectively binds to the cannabinoid type 2 (CB2) receptor, inhibiting adenylate cyclase activity and reducing intracellular cAMP levels. This leads to the suppression of NF‐κB‐mediated transcription of pro‐inflammatory cytokines, such as TNF‐α, IL‐1β, IL‐6, COX‐2, and iNOS. The results show notable anti‐inflammatory, analgesic, and antioxidant effects. Luteolin blocks the MAPK (ERK, JNK, p38) and NF‐κB signaling pathways by reducing IκBα degradation. This decrease lowers the production of inflammatory mediators, including COX‐2, iNOS, IL‐6, and TNF‐α. It also boosts cellular antioxidant defenses by scavenging ROS. Apigenin reduces NF‐κB nuclear translocation and inhibits STAT3 and MAPK pathways, resulting in decreased production of nitric oxide, prostaglandin E₂, and pro‐inflammatory cytokines. Additionally, apigenin induces Nrf2‐dependent antioxidant responses, which contribute to its powerful cytoprotective properties. Thus, all of these chemicals mutually control the oxidative stress and inflammatory pathways, establishing the molecular basis for the therapeutic advantages of *C. amboinicus* (Rusandi and Sadek 2024; Nguyen et al. 2020; Swamy et al. 2017; Muscolo et al. 2024; Barbosa et al. 2023; Adegbola et al. 2017; Amalia and Damanik 2018; Duraisamy et al. 2021).

The antibacterial effect is primarily attributed to carvacrol and thymol, which have been shown to act against bacteria by disrupting the lipid bilayer of the bacterial cell membrane, increasing permeability, allowing leakage of ATP and essential intracellular components, and resulting in cell lysis (Sabra et al. 2018; Sawant et al. 2023; Bhatt and Negi 2012). This membrane‐active mechanism of action is responsible for *C. amboinicus* essential oil's rapid efficacy in eliminating foodborne bacteria, implying its potential as a natural preservative for functional food applications (Dutra Da Silva et al. 2022; Gupta et al. 2016).

*C. amboinicus* has prebiotic and gut‐modulating characteristics due to its high soluble fiber and phenolic content. Dietary fiber is digested in the colon to create SCFAs, including acetate, propionate, and butyrate, which help the intestinal epithelial barrier function, reduce gut inflammation, and supply metabolic substrates for colonocytes (Adriani et al. 2019; Shubha and Bhatt 2015). To promote gut homeostasis and improve nutrient absorption, phenolic substances selectively increase the growth of beneficial microbiota (*Lactobacillus* and *Bifidobacterium*) while suppressing pathogenic strains (Yanza et al. 2022; Domínguez‐Avila et al. 2021; Kasprzak‐Drozd et al. 2021).

Together, these pathways demonstrate that *C. amboinicus'* multifunctional capabilities are based on molecular processes rather than actual biological consequences. Coordinated activities against antioxidant defense systems, inflammatory signaling, microbial membrane disruption, and microbiota metabolism provide solid scientific support for the use of *C. amboinicus* as a verified functional food and nutraceutical ingredient.

## 8 Challenges and Future Prospects

Despite considerable data supporting *C. amboinicus* ' nutritional and therapeutic potential, various scientific and commercial barriers continue to prevent its complete integration into the functional food and nutraceutical sectors. The high variability in phytochemical composition poses a major challenge, with significant differences in the levels of essential oils (thymol and carvacrol), rosmarinic acid, and flavonoids depending on geographic location, cultivation conditions, harvest stage, and extraction method. Such biochemical discrepancies threaten the consistency of biological activities and impede the development of standardized doses and formulation protocols. As a result, optimization techniques for agronomy, post‐harvest handling, and extraction procedures have been developed to ensure batch‐to‐batch consistency and quality control at industrial levels (Sabra et al. 2018; Ashaari et al. 2021; Silva et al. 2017; Arumugam et al. 2016; Kumar et al. 2020).

Another issue is the lack of human clinical evidence. *C. amboinicus* has demonstrated antioxidant, antibacterial, anti‐inflammatory, and gut‐modulating properties in vitro and in vivo; however, further research is required to assess the plant's efficacy and safety in humans. A therapeutic dosage range, pharmacokinetics, toxicity threshold, or even long‐term metabolic impact concerns that need to be addressed before the plant can receive regulatory approval for sale are not indicated by the available data. Controlled clinical studies are essential for progress in scientific and business fields by determining optimal intake levels, side effects, and functional outcomes for various groups (Paul et al. 2024; Raghavi et al. 2013; Shubha and Bhatt 2015; Arumugam et al. 2016; Solfaine et al. 2021).

Furthermore, regulatory obstacles hinder the widespread use and acceptance of *C. amboinicus* as a functional food ingredient. Comprehensive toxicological documentation and a precise specification defining safe application in drinks, dairy matrices, bread goods, and nutritional supplements are required by the current worldwide food safety recommendations (Granato et al. 2020; Serafini and Peluso 2017; MRF 2025). These are all now absent, which might discourage firms from making significant investments in product development. However, consumer awareness of *C. amboinicus* ' medicinal potential remains limited. The growing demand for natural, plant‐based health products will be met by creative forms such as protein shakes, quick mixes, fortified energy snacks, and ready‐to‐eat meals (Exporters India 2025; MRF 2025).

Future studies should focus on innovative technologies to enhance the bioavailability of phytochemicals from *C. amboinicus* , such as nanoencapsulation, microemulsion administration, and fermentation (Kim et al. 2022; Nguyen et al. 2020; Swamy et al. 2017). Another intriguing area of study is looking at its synergistic relationship with other functional elements, such as probiotics and dietary fiber, to produce novel nutraceuticals and functional meals that target gut health (Sabra et al. 2018; Adriani et al. 2019; Shubha and Bhatt 2015; Arumugam et al. 2016). Addressing these scientific, regulatory, and development obstacles will help *C. amboinicus* become an internationally recognized, evidence‐based ingredient for the functional food and health‐nutrition industries.

## 9 Conclusion

*C. amboinicus* is an emerging functional food ingredient with immense potential due to its rich nutritional profile and diverse bioactive properties. The presence of key phytochemicals such as thymol, carvacrol, and rosmarinic acid contributes to its antioxidant, anti‐inflammatory, and antimicrobial effects. These properties support its role in managing chronic diseases, including cardiovascular disorders, gastrointestinal conditions, and diabetes. Additionally, its potential as a natural food preservative offers a sustainable alternative to synthetic additives, aligning with the growing demand for clean‐label and eco‐friendly food products. However, several challenges hinder the full‐scale utilization of *C. amboinicus* in functional foods, including variability in bioactive compound content, limited clinical validation, and regulatory constraints. Addressing these issues through standardized extraction methods, bioavailability‐enhancing techniques such as nanoencapsulation, and rigorous clinical trials will be essential for establishing *C. amboinicus* as a scientifically validated functional ingredient. Future research should also explore the application of *C. amboinicus* in personalized nutrition, which could open new avenues for its inclusion in tailored dietary supplements. By overcoming these challenges, *C. amboinicus* has the potential to become a key player in the global health and nutrition industry, making a significant contribution to disease prevention and overall public health.

## Author Contributions

**Shani Upadhyay:** conceptualization (equal), investigation (equal), resources (equal), validation (equal), writing – original draft (equal), writing – review and editing (equal). **Rakesh Verma:** conceptualization (equal), supervision (equal), validation (equal), writing – review and editing (equal). **Shikha Gupta:** conceptualization (equal), investigation (equal), validation (equal), writing – review and editing (equal).

## Funding

The authors have nothing to report.

## Conflicts of Interest

The authors declare no conflicts of interest.

## Data Availability

The data presented in this study are available in the manuscript.

## References

[fsn371419-bib-0001] Adegbola, P. , I. Aderibigbe , W. Hammed , and T. Omotayo . 2017. “Antioxidant and Anti‐Inflammatory Medicinal Plants Have Potential Role in the Treatment of Cardiovascular Disease: A Review.” American Journal of Cardiovascular Diseases 7: 19–32. PMC5435602 28533927

[fsn371419-bib-0002] Adriani, A. , R. Asra , S. Novianti , and F. Fatati . 2019. “The Effect of *Coleus amboinicus* L. Supplementation on In Vitro Digestibility.” Pakistan Journal of Nutrition 18, no. 3: 241–246. 10.3923/pjn.2019.241.246.

[fsn371419-bib-0003] Al‐Elwany, O. A. A. I. , K. A. Hemida , M. A. Abdel‐Razek , et al. 2022. “Impact of Folic Acid in Modulating Antioxidant Activity, Osmoprotectants, Anatomical Responses, and Photosynthetic Efficiency of *Plectranthus amboinicus* Under Salinity Conditions.” Frontiers in Plant Science 13: 887091. 10.3389/fpls.2022.887091. 35968108 PMC9367479

[fsn371419-bib-0004] Amalia, N. R. , and M. R. M. Damanik . 2018. “Torbangun Leaves ( *Coleus amboinicus* Lour) Powder Capsules Supplementation Improve Lipid Profile and Blood Pressure in Men With Hypercholesterol.” Jurnal Gizi Dan Pangan 13, no. 2: 71–78. 10.25182/jgp.2018.13.2.71-78.

[fsn371419-bib-0005] Arumugam, G. , M. Swamy , and U. Sinniah . 2016. “ *Plectranthus amboinicus* (Lour.) Spreng: Botanical, Phytochemical, Pharmacological and Nutritional Significance.” Molecules 21, no. 4: 369. 10.3390/molecules21040369. 27043511 PMC6274163

[fsn371419-bib-0006] Ashaari, N. S. , M. H. Ab. Rahim , S. Sabri , et al. 2020. “Functional Characterization of a New Terpene Synthase From *Plectranthus amboinicus* .” PLoS One 15, no. 7: e0235416. 10.1371/journal.pone.0235416. 32614884 PMC7332032

[fsn371419-bib-0007] Ashaari, N. S. , N. E. Mohamad , A. H. Afzinizam , M.‐H. Ab Rahim , K. S. Lai , and J. O. Abdullah . 2021. “Chemical Composition of Hexane‐Extracted *Plectranthus amboinicus* Leaf Essential Oil: Maximizing Contents on Harvested Plant Materials.” Applied Sciences 11, no. 22: 10838. 10.3390/app112210838.

[fsn371419-bib-0008] Barbosa, M. D. O. , P. Wilairatana , G. M. L. Leite , et al. 2023. “Plectranthus Species With Anti‐Inflammatory and Analgesic Potential: A Systematic Review on Ethnobotanical and Pharmacological Findings.” Molecules 28, no. 15: 5653. 10.3390/molecules28155653. 37570622 PMC10419981

[fsn371419-bib-0009] Bathmanath, R. , Y. A. C. Yahya , M. M. Yusoff , and J. Vejayan . 2019. “Utilizing Coagulant Plants in the Development of Functional Dairy Foods and Beverages: A Mini Review.” Journal of Biological Sciences 19, no. 3: 259–271. 10.3923/jbs.2019.259.271.

[fsn371419-bib-0010] Bhatt, P. , G. S. Joseph , P. S. Negi , and M. C. Varadaraj . 2013. “Chemical Composition and Nutraceutical Potential of Indian Borage ( *Plectranthus amboinicus* ) Stem Extract.” Journal of Chemistry 2013, no. 1: 320329. 10.1155/2013/320329.

[fsn371419-bib-0011] Bhatt, P. , and P. S. Negi . 2012. “Antioxidant and Antibacterial Activities in the Leaf Extracts of Indian Borage ( *Plectranthus amboinicus* ).” Food and Nutrition Sciences 3, no. 2: 146–152. 10.4236/fns.2012.32022.

[fsn371419-bib-0012] Bibow, A. , and W. Oleszek . 2024. “Essential Oils as Potential Natural Antioxidants, Antimicrobial, and Antifungal Agents in Active Food Packaging.” Antibiotics 13, no. 12: 1168. 10.3390/antibiotics13121168. 39766558 PMC11672656

[fsn371419-bib-0013] Calderón Bravo, H. , N. Vera Céspedes , L. Zura‐Bravo , and L. A. Muñoz . 2021. “Basil Seeds as a Novel Food, Source of Nutrients and Functional Ingredients With Beneficial Properties: A Review.” Food 10, no. 7: 1467. 10.3390/foods10071467. PMC8303141 34202798

[fsn371419-bib-0014] Chakrabartty, I. , Y. K. Mohanta , A. Nongbet , et al. 2022. “Exploration of Lamiaceae in Cardio Vascular Diseases and Functional Foods: Medicine as Food and Food as Medicine.” Frontiers in Pharmacology 13: 894814. 10.3389/fphar.2022.894814. 35774598 PMC9237463

[fsn371419-bib-0015] Chandimali, N. , S. G. Bak , E. H. Park , et al. 2025. “Free Radicals and Their Impact on Health and Antioxidant Defenses: A Review.” Cell Death Discov 11, no. 1: 19. 10.1038/s41420-024-02278-8. 39856066 PMC11760946

[fsn371419-bib-0016] Damanik, R. 2009. “Torbangun ( *Coleus amboinicus* Lour): A Bataknese Traditional Cuisine Perceived as Lactagogue by Bataknese Lactating Women in Simalungun, North Sumatera, Indonesia.” Journal of Human Lactation 25, no. 1: 64–72. 10.1177/0890334408326086. 18984829

[fsn371419-bib-0017] Domínguez‐Avila, J. A. , J. A. Villa‐Rodriguez , M. Montiel‐Herrera , et al. 2021. “Phenolic Compounds Promote Diversity of Gut Microbiota and Maintain Colonic Health.” Digestive Diseases and Sciences 66, no. 10: 3270–3289. 10.1007/s10620-020-06676-7. 33111173

[fsn371419-bib-0018] Donno, D. , S. Hassani , T. Sofoini , et al. 2021. “Traditional Foods and Sustainable Rural Development: Exploiting the Case of the Comoros Tea as a Potential Source of Bioactive Compounds.” Sustainability 13, no. 11: 5815. 10.3390/su13115815.

[fsn371419-bib-0019] Duraisamy, P. , B. Manikandan , A. Koodalingam , A. Munusamy , and M. Ramar . 2021. “Anti‐Inflammatory, Anti‐Nociceptive and Anti‐Oxidant Activities of Carvacrol Containing Leaf Extracts of Edible Indian Borage Plant *Plectranthus amboinicus* : An In Vivo and In Vitro Approach.” Comparative Clinical Pathology 30, no. 3: 397–413. 10.1007/s00580-021-03230-3.

[fsn371419-bib-0020] Dutra Da Silva, B. , P. C. Bernardes , P. F. Pinheiro , J. Di Giorgio Giannotti , and C. D. Roberto . 2022. “ *Plectranthus amboinicus* (Lour.) Spreng. Essential Oil as a Natural Alternative for the Conservation of Beef Patties Stored Under Refrigeration.” Food Bioscience 49: 101896. 10.1016/j.fbio.2022.101896.

[fsn371419-bib-0021] El‐hawary, S. S. , R. H. El‐sofany , A. R. Abdel‐Monem , and R. S. Ashour . 2012. “Phytochemical Screening, DNA Fingerprinting, and Nutritional Value of *Plectranthus amboinicus* (Lour.) Spreng.” Pharmacognosy Journal 4, no. 30: 10–13. 10.5530/pj.2012.30.2.

[fsn371419-bib-0022] El‐hawary, S. S. , R. H. El‐sofany , A. R. Abdel‐Monem , R. S. Ashour , and A. A. Sleem . 2012. “Polyphenolics Content and Biological Activity of *Plectranthus amboinicus* (Lour.) Spreng Growing in Egypt (Lamiaceae).” Pharmacognosy Journal 4, no. 32: 45–54. 10.5530/pj.2012.32.9.

[fsn371419-bib-0023] Exporters India . 2025. “Indian Borage Leaf Power, *Coleus Amboinicus* , Style: Dried at Rs 989 in Chennai—ID: 5419829.” https://www.exportersindia.com/product‐detail/25kg‐100‐gms‐indian‐borage‐leaf‐power‐coleus‐amboinicus‐5419829.htm.

[fsn371419-bib-0024] Food Struct . 2025. Basil Nutrition: Calories, Carbs, GI, Protein, Fiber, Fats. Food Struct. https://foodstruct.com/food/basil.

[fsn371419-bib-0025] Fujiana, F. , K. Gres , Y. S. Muttalib , A. Salam , I. W. Wirawanti , and D. Fadly . 2021. “Fresh Noodles Enriched With *Coleus amboinicus* Lour Leaves to Lower the Premenstrual Syndrome Level.” IOP Conf Ser Earth Environ Sci 807, no. 2: 022064. 10.1088/1755-1315/807/2/022064.

[fsn371419-bib-0026] Granato, D. , F. J. Barba , D. Bursać Kovačević , J. M. Lorenzo , A. G. Cruz , and P. Putnik . 2020. “Functional Foods: Product Development, Technological Trends, Efficacy Testing, and Safety.” Annual Review of Food Science and Technology 11, no. 1: 93–118. 10.1146/annurev-food-032519-051708. 31905019

[fsn371419-bib-0027] Gupta, S. K. , P. S. Negi , and Fruit and Vegetable Technology Department, CSIR‐Central Food Technological Research Institute . 2016. “Antibacterial Activity of Indian Borage ( *Plectranthus amboinicus* Benth) Leaf Extracts in Food Systems and Against Natural Microflora in Chicken Meat.” Food Technology and Biotechnology 54: 90–102. 10.17113/ftb.54.01.16.3973. 27904397 PMC5105625

[fsn371419-bib-0028] Healthline . 2025. Spinach 101: Nutrition Facts and Health Benefits. Healthline. https://www.healthline.com/nutrition/foods/spinach.

[fsn371419-bib-0029] Hullatti, K. , and P. Bhattacharjee . 2011. “Pharmacognostical Evaluation of Different Parts of *Coleus amboinicus* Lour., Lamiaceae.” Pharmacognosy Journal 3, no. 24: 39–44. 10.5530/pj.2011.24.8.

[fsn371419-bib-0030] Hunthayung, K. , and S. Bhawamai . 2025. “Nutritional Profiles of Moringa Pods ( *Moringa oleifera* ) and Their Extract Activities on SaOS‐2 Osteoblast Cells.” Bioactive Compounds in Health and Disease 8: 217–230. 10.31989/bchd.v8i5.1608.

[fsn371419-bib-0083] ICAR‐IIHR . 2021. Bengaluru organizes National Horticulture Fair ICAR. https://icar.org.in/en/icar‐iihr‐bengaluru‐organizes‐national‐horticulture‐fair‐2021.

[fsn371419-bib-0031] Islam, Z. , S. M. R. Islam , F. Hossen , K. Mahtab‐ul‐Islam , M. R. Hasan , and R. Karim . 2021. “ *Moringa oleifera* Is a Prominent Source of Nutrients With Potential Health Benefits.” International Journal of Food Science 2021: 1–11. 10.1155/2021/6627265. PMC8373516 34423026

[fsn371419-bib-0032] Kasprzak‐Drozd, K. , T. Oniszczuk , M. Stasiak , and A. Oniszczuk . 2021. “Beneficial Effects of Phenolic Compounds on Gut Microbiota and Metabolic Syndrome.” International Journal of Molecular Sciences 22, no. 7: 3715. 10.3390/ijms22073715. 33918284 PMC8038165

[fsn371419-bib-0033] Kim, J. H. , D. H. Kim , S. Jo , et al. 2022. “Immunomodulatory Functional Foods and Their Molecular Mechanisms.” Experimental & Molecular Medicine 54, no. 1: 1–11. 10.1038/s12276-022-00724-0. 35079119 PMC8787967

[fsn371419-bib-0034] Koti, B. , A. Gore , A. H. M. Thippeswamy , A. H. M. Viswanatha Swamy , and R. Kulkarni . 2011. “Alcoholic Leaf Extract of *Plectranthus amboinicus* Regulates Carbohydrate Metabolism in Alloxan‐Induced Diabetic Rats.” Indian Journal of Pharmacology 43, no. 3: 286–290. 10.4103/0253-7613.81520. 21713092 PMC3113380

[fsn371419-bib-0035] Kumar, P. , S. Singh , and N. Kumar . 2020. “ *Plectranthus amboinicus* : A Review on Its Pharmacological and, Pharmacognostical Studies.” American Journal of Physiology, Biochemistry and Pharmacology 10, no. 2: 55. 10.5455/ajpbp.20190928091007.

[fsn371419-bib-0036] Laila, F. , D. Fardiaz , N. D. Yuliana , M. R. M. Damanik , and F. N. A. Dewi . 2020. “Methanol Extract of *Coleus amboinicus* (Lour) Exhibited Antiproliferative Activity and Induced Programmed Cell Death in Colon Cancer Cell WiDr.” International Journal of Food Science 2020: 1–12. 10.1155/2020/9068326. PMC7003269 32047805

[fsn371419-bib-0037] Lekshmi, R. G. K. , K. Jayathilakan , K. Sarika , et al. 2019. “Effect of *Plectranthus amboinicus* Leaf Extract on the Quality Attributes of Microencapsulated Fish Oil Fortified Soup Powder.” Fishery Technology 56, no. 4: 94973. 10.56093/ft.v56i4.94973.

[fsn371419-bib-0038] Liu, Y.‐M. , C. Liu , Y. S. Deng , et al. 2025. “Beneficial Effects of Dietary Herbs on High‐Fat Diet‐Induced Obesity Linking With Modulation of Gut Microbiota.” Food Mediine Homology 2, no. 2: 9420034. 10.26599/FMH.2025.9420034.

[fsn371419-bib-0039] Michel, J. , N. Z. Abd Rani , and K. Husain . 2020. “A Review on the Potential Use of Medicinal Plants From Asteraceae and Lamiaceae Plant Family in Cardiovascular Diseases.” Frontiers in Pharmacology 11: 852. 10.3389/fphar.2020.00852. 32581807 PMC7291392

[fsn371419-bib-0040] Mirwandhono, R. E. , Y. Janviktor , and B. A. E. A. Kaban . 2023. “Chemical and In Vitro Evaluation of Biscuit of Bangun‐Bangun Leaf ( *Coleus amboinicus* Lour).” IOP Conf Ser Earth Environ Sci 1286, no. 1: 012015. 10.1088/1755-1315/1286/1/012015.

[fsn371419-bib-0041] Morales‐Payan, J. P. 2006. “Growth of Aromatic Coleus ( *Coleus amboinicus* Lour.) as Affected by Biostimulators.”

[fsn371419-bib-0042] Moyeenudin, H. M. , and R. Thiruchelvi . 2021. “The Antiviral and Antibacterial Properties of *Plectranthus amboinicus* and *Piper longum* With the Addition to Focaccia Bread Nutritional Value and Sensory Evaluation.” Research Journal of Pharmacy and Technology 14, no. 9: 4951–4956. 10.52711/0974-360X.2021.00861.

[fsn371419-bib-0043] MRF . 2025. “Coleus and Turmeric Market Size, Industry Share, Growth, Forecast‐2035.” https://www.marketresearchfuture.com/reports/coleus‐and‐turmeric‐market‐39036.

[fsn371419-bib-0044] Muscolo, A. , O. Mariateresa , T. Giulio , and R. Mariateresa . 2024. “Oxidative Stress: The Role of Antioxidant Phytochemicals in the Prevention and Treatment of Diseases.” International Journal of Molecular Sciences 25, no. 6: 3264. 10.3390/ijms25063264. 38542238 PMC10970659

[fsn371419-bib-0045] My Food Data . 2025a. Nutrition Facts for Fresh Peppermint. My Food Data. https://tools.myfooddata.com/nutrition‐facts/173474/wt1.

[fsn371419-bib-0046] My Food Data . 2025b. Nutrition Facts for Spinach Raw. My Food Data. https://tools.myfooddata.com/nutrition‐facts/342205/100g.

[fsn371419-bib-0047] Nababan, D. 2018. “Analysis of Nutritional Content of Torbangun ( *Coleus amboinicus* Lour) Leaf Biscuit.” Proceedings of the International Conference on Applied Science and Health 3: 299–304.

[fsn371419-bib-0048] Nasution, S. S. , R. Eliana , E. Aizar , and R. Pramita . 2022. “Effectiveness of *Coleus amboinicus* Consumption Interventions in Increasing Breast Milk Production and Improving Maternal Health Status During COVID 19 Pandemic.” Open Access Macedonian Journal of Medical Sciences 10: 202–208. 10.3889/oamjms.2022.7094.

[fsn371419-bib-0049] Nguyen, N. Q. , L. V. Minh , L. H. Trieu , et al. 2020. “Evaluation of Total Polyphenol Content, Total Flavonoid Content, and Antioxidant Activity of *Plectranthus amboinicus* Leaves.” IOP Conference Series: Materials Science and Engineering 736: 062017. 10.1088/1757-899X/736/6/062017.

[fsn371419-bib-0050] Ngyon, C. H. , and W. T. Hlaing . 2025. “Study on the Phytochemical Constituents, Elemental Analysis and Antimicrobial Activities From the Leaves of *Coleus amboinicus* Lour. ( Zi Yar).”

[fsn371419-bib-0051] NutrientOptimiser . 2025. Nutrition Facts for Peppermint, Recommended Daily Values and Analysis. NutrientOptimiser. https://nutrientoptimiser.com/nutritional‐value‐peppermint‐fresh.

[fsn371419-bib-0052] Paul, K. , B. H. J. Gowda , U. Hani , et al. 2024. “Traditional Uses, Phytochemistry, and Pharmacological Activities of Coleusamboinicus: A Comprehensive Review.” Current Pharmaceutical Design 30, no. 7: 519–535. 10.2174/0113816128283267240130062600. 38321896

[fsn371419-bib-0053] Phattayanon, N. , A. Assawamakin , P. Dechwongya , and A. Dadookel . 2024. “From Tradition to Therapy: *Plectranthus amboinicus* as a Remedy for Respiratory Inflammation.” International Journal of Health Sciences 22: 0491.

[fsn371419-bib-0054] Phattayanon, N. , A. Assawamakin , P. Dechwongya , and A. Dadookel . 2025. “From Tradition to Therapy: *Plectranthus amboinicus* as a Remedy for Respiratory Inflammation.” Interprofessional Journal of Health Sciences 24: 491.

[fsn371419-bib-0055] Raghavi, S. , V. G. Karthigeyan , and S. Prabhu . 2013. “Protective Effect of *Plectranthus amboinicus* Leaf Extract Containing Luteolin Flavanoid in Isoproterenol Hydrochloride Induced Myocardial Infarction in Rats—An Assessment on Biochemical and Cellular Changes.” Research Journal of Engineering and Technology 4: 195–198.

[fsn371419-bib-0056] Rajkumar, M. , D. Kirubakaran , K. Selvam , et al. 2024. “Green Synthesis of Gelatin‐Loaded Magnesium Hydroxide Nanocomposite Biomaterial Using *Coleus amboinicus* Leaf Extract for Enhanced Antibacterial, Antioxidant, Anticholinergic, and Wound Healing Activities.” Journal of Materials Research 39, no. 4: 548–564. 10.1557/s43578-023-01249-6.

[fsn371419-bib-0057] Rasineni, G. , D. Siddavattam , and A. R. Reddy . 2025. Free Radical Quenching Activity and Polyphenols in Three Species of Coleus. ResearchGate. https://www.researchgate.net/publication/228673272_Free_radical_quenching_activity_and_polyphenols_in_three_species_of_Coleus.

[fsn371419-bib-0058] Rohini, R. M. , and G. R. Smitha . 2021. “Glossary on Medicinal Herbs Bulletin 2021.” Medicinal Plants 2: 96965. 10.13140/RG.2.2.33186.96965.

[fsn371419-bib-0059] Roy, S. , and S. Dhaneshwar . 2023. “Role of Prebiotics, Probiotics, and Synbiotics in Management of Inflammatory Bowel Disease: Current Perspectives.” World Journal of Gastroenterology 29, no. 14: 2078–2100. 10.3748/wjg.v29.i14.2078. 37122604 PMC10130969

[fsn371419-bib-0060] Rusandi, F. S. , and N. F. Sadek . 2024. “Comparison of Different Methods for Evaluating the Antioxidant Activity of Instant Torbangun ( *Coleus amboinicus* L.) Drinks.” IOP Conference Series: Earth Environmental Sciences 1324, no. 1: 012124. 10.1088/1755-1315/1324/1/012124.

[fsn371419-bib-0061] Sabra, A. S. , T. Astatkie , A. Alataway , et al. 2018. “Response of Biomass Development, Essential Oil, and Composition of *Plectranthus amboinicus* (Lour.) Spreng. To Irrigation Frequency and Harvest Time.” Chemistry & Biodiversity 15, no. 3: e1800005. 10.1002/cbdv.201800005. 29393581

[fsn371419-bib-0062] Sajimin, S. , N. D. Purwantari , E. Sutedi , and O. Oyo . 2025. “Effect of Cutting Interval to Productivity and Quality of Bangun‐Bangun ( *Coleus amboinicus* L.) as a Forage Promising Commodity.” 17.

[fsn371419-bib-0063] Santos Filipe, M. , G. Bangay , F. Z. Brauning , et al. 2025. “ *Plectranthus amboinicus* : A Systematic Review of Traditional Uses, Phytochemical Properties, and Therapeutic Applications.” Pharmaceuticals 18, no. 5: 707. 10.3390/ph18050707. 40430526 PMC12114729

[fsn371419-bib-0064] Saqib, S. , F. Ullah , M. Naeem , et al. 2022. “Mentha: Nutritional and Health Attributes to Treat Various Ailments Including Cardiovascular Diseases.” Molecules 27, no. 19: 6728. 10.3390/molecules27196728. 36235263 PMC9572119

[fsn371419-bib-0065] Sawant, S. , T. C. Baldwin , O. Metryka , and A. Rahman . 2023. “Evaluation of the Effect of *Plectranthus amboinicus* L. Leaf Extracts on the Bacterial Antioxidant System and Cell Membrane Integrity of *Pseudomonas aeruginosa* PA01 and *Staphylococcus aureus* NCTC8325.” Pathogens 12, no. 6: 853. 10.3390/pathogens12060853. 37375543 PMC10302856

[fsn371419-bib-0066] Serafini, M. , and I. Peluso . 2017. “Functional Foods for Health: The Interrelated Antioxidant and Anti‐Inflammatory Role of Fruits, Vegetables, Herbs, Spices and Cocoa in Humans.” Current Pharmaceutical Design 22, no. 44: 6701–6715. 10.2174/1381612823666161123094235. PMC5427773 27881064

[fsn371419-bib-0067] Shubha, J. R. , and P. Bhatt . 2015. “ *Plectranthus amboinicus* Leaves Stimulate Growth of Probiotic *L. plantarum* : Evidence for Ethnobotanical Use in Diarrhea.” Journal of Ethnopharmacology 166: 220–227. 10.1016/j.jep.2015.02.055. 25796406

[fsn371419-bib-0068] Silitonga, M. , S. Ilyas , S. Hutahaean , and H. Sipahutar . 2014. “Levels of Apigenin and Immunostimulatory Activity of Leaf Extracts of Bangunbangun ( *Plectranthus Amboinicus* Lour).” International Journal of Biology 7, no. 1: p46. 10.5539/ijb.v7n1p46.

[fsn371419-bib-0069] Silva, S. T. , S. K. V. Bertolucci , S. H. B. Da Cunha , L. E. S. Lazzarini , M. C. Tavares , and J. E. B. P. Pinto . 2017. “Effect of Light and Natural Ventilation Systems on the Growth Parameters and Carvacrol Content in the In Vitro Cultures of *Plectranthus amboinicus* (Lour.) Spreng.” Plant Cell, Tissue and Organ Culture 129, no. 3: 501–510. 10.1007/s11240-017-1195-6.

[fsn371419-bib-0070] Ślusarczyk, S. , A. Cieślak , Y. R. Yanza , et al. 2021. “Phytochemical Profile and Antioxidant Activities of *Coleus amboinicus* Lour. Cultivated in Indonesia and Poland.” Molecules 26, no. 10: 2915. 10.3390/molecules26102915. 34068950 PMC8156032

[fsn371419-bib-0071] Solfaine, R. , L. Muniroh , S. Sadarman , A. Apriza , and A. Irawan . 2021. “Anti‐Inflammatory Effect of *Coleus amboinicus* Leaves Extract on Uric Acid‐Induced Nephrotoxicity Rats.” Advances in Animal and Veterinary Sciences 9, no. 10: 1558. 10.17582/journal.aavs/2021/9.10.1553.1558.

[fsn371419-bib-0072] Sun‐Waterhouse, D.‐X. , X.‐Y. Chen , Z.‐H. Liu , G. I. N. Waterhouse , and W.‐Y. Kang . 2024. “Transformation From Traditional Medicine‐Food Homology to Modern Food‐Medicine Homology.” Food Mediine Homology 1, no. 1: 9420014. 10.26599/FMH.2024.9420014.

[fsn371419-bib-0073] Suryowati, T. , and M. Gultom . 2019. “Effect of Torbangun ( *Coleus amboinicus* Lour) on Blood Pressure in Women With Hypercholesterolemia.” Journal of Physics Conference Series 1146: 012002. 10.1088/1742-6596/1146/1/012002.

[fsn371419-bib-0074] Swamy, M. K. , G. Arumugam , R. Kaur , A. Ghasemzadeh , M. Yusoff , and U. R. Sinniah . 2017. “GC‐MS Based Metabolite Profiling, Antioxidant and Antimicrobial Properties of Different Solvent Extracts of Malaysian *Plectranthus amboinicus* Leaves.” Evidence‐Based Complementary and Alternative Medicine 2017, no. 1: 1517683. 10.1155/2017/1517683. 28424737 PMC5382359

[fsn371419-bib-0075] Tan, B. L. , M. E. Norhaizan , W.‐P.‐P. Liew , and H. Sulaiman Rahman . 2018. “Antioxidant and Oxidative Stress: A Mutual Interplay in Age‐Related Diseases.” Frontiers in Pharmacology 9: 1162. 10.3389/fphar.2018.01162. 30405405 PMC6204759

[fsn371419-bib-0076] Thirugnanasampandan, R. , G. Ramya , M. Gogulramnath , R. Jayakumar , and M. S. Kanthimathi . 2014. “Evaluation of Cytotoxic, DNA Protecting and LPS Induced MMP‐9 Down Regulation Activities of *Plectranthus amboinicus* (Lour) Spreng. Essential Oil.” Pharmacognosy Journal 7, no. 1: 32–36. 10.5530/pj.2015.7.3.

[fsn371419-bib-0077] Thiruvengadam, S. 2025. Comparative Study on Free Radical Ameliorating Potential of Stem and the Leaf Extracts of *Plectranthus amboinicus* . ResearchGate. https://www.researchgate.net/publication/380824747_Comparative_study_on_free_radical_ameliorating_potential_of_stem_and_the_leaf_extracts_of_Plectranthus_amboinicus.

[fsn371419-bib-0078] USDA FoodData Central . 2025. Food Search. USDA FoodData Central. https://fdc.nal.usda.gov/food‐search?component=1185.

[fsn371419-bib-0079] Wadikar, D. D. , and P. E. Patki . 2016. “Coleus Aromaticus: A Therapeutic Herb With Multiple Potentials.” Journal of Food Science and Technology 53, no. 7: 2895–2901. 10.1007/s13197-016-2292-y. 27765960 PMC5052183

[fsn371419-bib-0080] Waseem, M. , S. Akhtar , M. F. Manzoor , et al. 2021. “Nutritional Characterization and Food Value Addition Properties of Dehydrated Spinach Powder.” Food Science & Nutrition 9, no. 2: 1213–1221. 10.1002/fsn3.2110. 33598205 PMC7866621

[fsn371419-bib-0081] Yanza, Y. R. , M. Szumacher‐Strabel , D. Lechniak , et al. 2022. “Dietary *Coleus amboinicus* Lour. Decreases Ruminal Methanogenesis and Biohydrogenation, and Improves Meat Quality and Fatty Acid Composition in Longissimus Thoracis Muscle of Lambs.” Journal of Animal Science and Biotechnology 13, no. 1: 5. 10.1186/s40104-021-00654-3. 35027089 PMC8765733

[fsn371419-bib-0082] Yu, Y.‐Y. , H. Q. Fu , H. Y. du , et al. 2025. “Overview of Research on the Application of Medicine Food Homologous Bioactive Ingredients to Functional Constipation.” Food Mediine Homology 2, no. 4: 9420057. 10.26599/FMH.2025.9420057.

